# Meta‐analysis of the procedural risks of carotid endarterectomy and carotid artery stenting over time

**DOI:** 10.1002/bjs.10717

**Published:** 2017-12-04

**Authors:** K. Lokuge, D. D. de Waard, A. Halliday, A. Gray, R. Bulbulia, B. Mihaylova

**Affiliations:** ^1^ Health Economics Research Centre, University of Oxford Oxford UK; ^2^ Medical Research Council Population Health Research Unit, Clinical Trial Service Unit and Epidemiological Studies Unit, Nuffield Department of Population Health University of Oxford Oxford UK; ^3^ Nuffield Department of Surgical Sciences University of Oxford Oxford UK; ^4^ Department of Vascular Surgery University Medical Centre Utrecht Utrecht The Netherlands

## Abstract

**Background:**

Stroke/death rates within 30 days of carotid endarterectomy (CEA) and carotid artery stenting (CAS) in RCTs inform current clinical guidelines. However, the risks may have changed in recent years with wider use of effective stroke prevention therapies, especially statins, improved patient selection and growing operator expertise. The aim of this study was to investigate whether the procedural stroke/death risks from CEA and CAS have changed over time.

**Methods:**

MEDLINE and Embase were searched systematically from inception to May 2016 for observational cohort studies of CEA and CAS. Studies included reported on more than 1000 patients, with 30‐day outcomes after the procedure according to patients' symptom status (recent stroke or transient ischaemic attack). Restricted maximum likelihood random‐effects and meta‐regressions methods were used to synthesize procedural stroke/death rates of CEA and CAS according to year of study recruitment completion.

**Results:**

Fifty‐one studies, including 223 313 patients undergoing CEA and 72 961 undergoing CAS, were reviewed. Procedural stroke/death risks of CEA decreased over time in symptomatic and asymptomatic patients. Risks were substantially lower in studies completing recruitment in 2005 or later, both in symptomatic (5·11 per cent before 2005 versus 2·68 per cent from 2005 onwards; P = 0·002) and asymptomatic (3·17 versus 1·50 per cent; P < 0·001) patients. Procedural stroke/death rates of CAS did not change significantly over time (4·77 per cent among symptomatic and 2·59 per cent among asymptomatic patients). There was substantial heterogeneity in event rates and recruitment periods were long.

**Conclusions:**

Risks of procedural stroke/death following CEA appear to have decreased substantially. There was no evidence of a change in stroke/death rates following CAS.

## Abstract 2

Endarterectomy outcomes improving

## Introduction

Stroke is a major cause of death and disability in the developed world^1^, ^2^ and carotid artery stenosis is one of the main causes of ischaemic stroke. RCTs have shown that carotid endarterectomy (CEA) has early (procedural) hazards, but is an effective long‐term stroke prevention treatment, in both symptomatic (patients who have recently had a stroke or transient ischaemic attack (TIA))^3^, ^4^ and asymptomatic^5^ patients. More recently, carotid artery stenting (CAS) has been introduced. RCTs^6^, ^7^, ^8^ comparing these procedures have shown that CAS has higher procedural stroke/death risks than CEA in symptomatic patients, whereas there was no significant difference in procedural stroke/death risks of the two procedures in asymptomatic patients^7^, ^9^.

Clinical guidelines and cost‐effectiveness analyses of CEA and CAS are based on data from RCTs, although the patients in these trials were mostly recruited some years ago, before 2005. International guidelines^10^, ^11^ recommend CEA for suitable symptomatic patients with a tight carotid stenosis (more than 50–70 per cent narrowed, by diameter) provided that the surgical procedural stroke/death risk is less than 6 per cent. For asymptomatic patients with carotid artery stenosis over 60 per cent, guidelines suggest that CEA may be considered if the procedural stroke/death risk is less than 3 per cent. For CAS, less specific complication rates are specified, as CAS may sometimes be reserved for patients who are high risk for CEA because of severe co‐morbidities or unusual vascular anatomical characteristics^12^. Although these recommendations are usually based on level I evidence from RCTs, procedural risk may have changed since these trials were conducted.

Control of vascular risk factors has intensified with the widespread use of statins after 2005, and this is also likely to have contributed to reductions in procedural risks^5^, ^13^. Patient selection for CEA and CAS has also improved^14^, with patients considered unfavourable for CEA owing to hostile anatomy or co‐morbidities being referred to CAS. Emerging evidence suggests that in‐hospital stroke/death risks of CEA and CAS, and stroke/death rates within 30 days of the procedure in asymptomatic patients undergoing CEA may have decreased^15^, ^16^.

## Methods

### Study selection

PRISMA guidelines^17^ were followed. MEDLINE and Embase were searched from inception to 20 May 2016 for observational cohort studies reporting procedural risks in patients undergoing CEA and CAS. A combination of terms related to the presence of carotid stenosis, CEA and CAS interventions, and outcomes made up the core search criteria, which were then combined with terms identifying observational cohort studies (Appendix S1, supporting information). Articles were screened by title and abstract, followed by full‐text review to identify observational cohort studies with more than 1000 patients diagnosed with carotid stenosis who had undergone CEA or CAS. Studies were considered only if they reported outcomes by patients' symptom status (recent history of stroke or TIA) and if they had a follow‐up period of at least 30 days after the procedure. Studies also had to report at least one of the following procedural outcomes: stroke, death, myocardial infarction (MI), subcategories of stroke, or a composite endpoint of any of these outcomes. When study populations overlapped across eligible articles, only the most recent one was included. The reference lists of recent systematic reviews^18^, ^19^, ^20^, ^21^ were checked for further relevant studies.

### Quality assessment

An adapted version of the Newcastle quality assessment scale^22^ was used to evaluate risk of bias (Appendix S2, supporting information) based on patient selection (representativeness, description of risk factors, ascertainment of exposure) and outcome evaluation (method of outcome assessment, length and adequacy of follow‐up). The maximum score was 8, with higher scores indicating better study quality.

Procedural outcomes were defined as events occurring within 30 days from the procedure. The primary procedural outcome was stroke or death. Further outcomes of interest included death, stroke, MI, stroke/death/MI, major stroke (modified Rankin score at least 3) and minor stroke (modified Rankin score less than 3).

Procedural outcomes were summarized by participants' symptom status at study entry. Event rates were also summarized according to whether the study recruitment period ended before 2005, or thereafter, because of the substantially increased use of statins and other stroke prevention treatments in later years.

### Partial second review

One author reviewed all citations. A second author screened a random sample of 20 per cent of the citations^17^ by title and abstract, and then by full text. The second reviewer also assessed the risk of bias and extracted data from 20 per cent of the included studies. The results were compared for concordance with those of the first reviewer.

### Statistical analysis

The restricted maximum likelihood random‐effects method^23^ with Freeman–Tukey (double arcsine) transformation^24^, ^25^ was used to synthesize event rates across observational cohort studies. The analyses were executed in R Studio v0.99.903 (R Consortium, Boston, Massachusetts, USA), with package Metaphor version 1.9‐9. Heterogeneity between studies was assessed using P from the I ^2^ heterogeneity statistic^26^, and the difference between pooled estimates of studies with recruitment before 2005 or from 2005 onwards was assessed using two‐sided t tests, after logarithmic transformation of event rates. A 95 per cent level of significance was used. Random‐effects logistic meta‐regressions^27^ were performed with year of completion of study recruitment as an explanatory variable to assess annual time trends in procedural event rates.

Studies with a quality assessment score of less than 6 were excluded in a sensitivity analysis. Observational cohort studies were also further stratified by method of outcome assessment (whether assessed by an independent neurologist or not), and by geographical region (Europe or North America), which had been identified as possible sources of heterogeneity.

## Results

### Study selection

**Figure 1:**
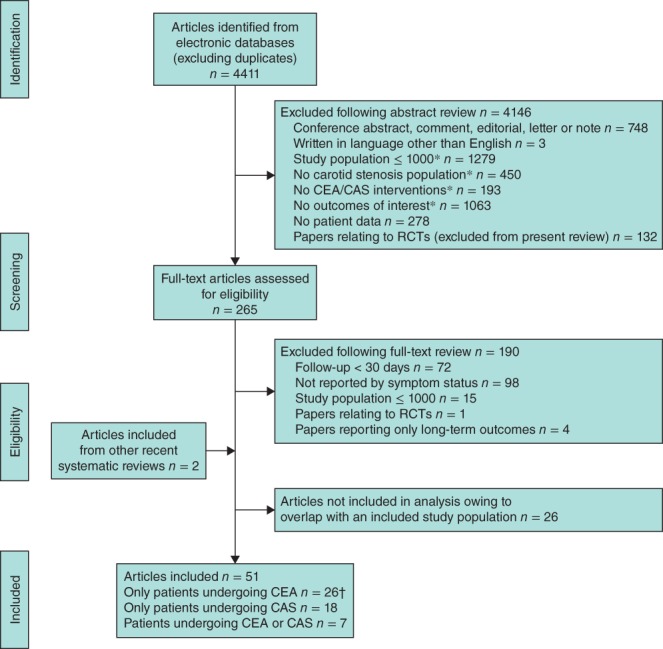
Selection of studies for inclusion in the systematic review. *Citations under these criteria were included at first category, but may have also qualified for further exclusion criteria. †Vikatmaa and colleagues^28^ contributed data for eight separate study populations; Kresowik and co‐workers^29^ contributed data for two separate study populations. CEA, carotid endarterectomy; CAS, carotid artery stenting

Some 4411 citations were screened by title and abstract by the first reviewer, of which 265 progressed to full‐text review (Fig. 1). Seventy‐five articles fulfilled the eligibility criteria, but 26 of these had study populations overlapping with those in other eligible studies (Table S1 and Appendix S3, supporting information). Two further articles were added following review of references from recent reviews. The second reviewer screened 883 manuscripts (20·0 per cent) by title and abstract. There was 98 per cent concordance between the two reviewers in the judgement of eligibility of studies; any disagreement was resolved by discussion. Fifty‐one articles (Table S2 and Appendix S3, supporting information) were included in the review, and underwent quality assessment and data extraction. These studies included 223 313 patients undergoing CEA and 72 961 undergoing CAS.

### Study quality assessment

The mean quality score of included studies was 6·6 (*Table S3*, supporting information). Eight studies were scored down by 1 point because the study population was not representative of usual carotid stenosis populations, either because the average age of the cohort was above 75 years or population characteristics were not reported. Eighteen studies were scored down because of lack of a description of the definition of ‘symptomatic’ patients. When explicit, the majority of studies defined symptomatic patients as those who had a stroke or TIA within 6 months before the procedure. However, larger retrospective databases defined symptomatic patients as those experiencing ‘stroke or TIA any time before procedure’. Twenty‐one studies were scored down owing to their method of outcome assessment. Although outcome assessment by independent neurologists is the standard, studies reporting such assessment are rare. Therefore, studies in which outcomes were assessed by record linkage, or by consultant neurologists/surgeons or trained nurses but not by an independent neurologist, received the highest score on this indicator; the score was reduced where outcomes were self‐reported by the patient or the method of outcome assessment was not stated, The second reviewer also extracted data from ten articles (20 per cent of those included in the systematic review) and assessed their risk of bias, with results matching those of the first reviewer.

Procedural stroke/death was the most commonly reported outcome, reported in 29 CEA studies (8 before 2005, 21 from 2005 onwards) and 13 CAS studies (2 and 11 respectively). The period of study recruitment ranged from 1976 to 2014 in CEA studies, and from 1989 to 2011 in CAS studies. Only seven CEA studies and five CAS studies reported procedural MI event rates. Three CEA studies and seven CAS studies reported results for major and minor strokes separately.

### Procedural risks of carotid endarterectomy: symptomatic patients

**Figure 2:**
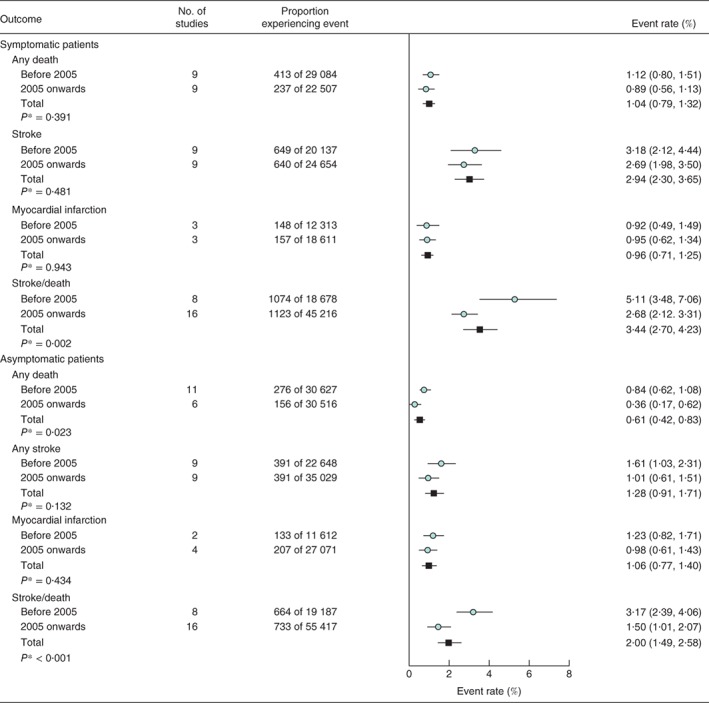
Carotid endarterectomy procedural risks in patients classified by symptom status. Adverse event rates are summarized across all studies and separately for those completing recruitment before 2005 or from 2005 onwards. Event rates are shown with 95 per cent confidence intervals. *Before 2005 versus 2005 onwards (2‐sided t test)

**Figure 3:**
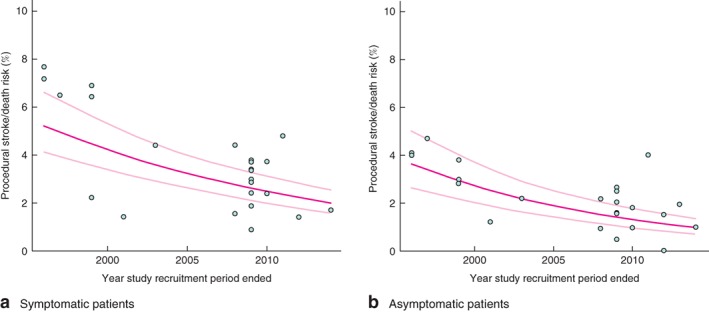
Meta‐regression of carotid endarterectomy procedural stroke/death rates among **a** symptomatic and **b** asymptomatic patients. Risks are shown with 95 per cent confidence intervals

The procedural stroke/death risk following CEA in symptomatic patients across 24 studies was 3·44 (95 per cent c.i. 2·70 to 4·23) per cent (*Fig*. 2). However, the procedural stroke/death risk in studies where the recruitment period ended in 2005 or later was significantly lower than in studies completing recruitment before 2005: 2·68 (2·12 to 3·31) *versus* 5·11 (3·48 to 7·06) per cent (*P* = 0·002) (*Fig*. 2). Substantial heterogeneity in rates was observed across individual studies (*Fig. S1*, supporting information). The meta‐regression analysis assessing annual trends over time indicated a 6·1 (95 per cent c.i. 3·0 to 9·2) per cent per annum reduction in CEA procedural stroke/death rate in symptomatic patients (*Fig*. 3 *a*). When considered separately, the rates of procedural deaths and strokes were lower in later studies, but the differences were not statistically significant (*Fig*. 2), and data on procedural major and minor strokes separately were limited (*Fig. S2*, supporting information). There was no statistically significant difference between rates of procedural MI in studies completing recruitment before 2005 and later studies (*P* = 0·943); the overall risk was 0·96 (0·71 to 1·25) per cent (*Fig*. 2).

Three studies^30^, ^31^, ^32^ in the review reported procedural outcomes by timing of procedure after development of symptoms, suggesting higher procedural stroke/death rates with an earlier procedure, but mixed results regarding the time interval over which procedural risks might be increased. Two studies^30^, ^33^ reported procedural outcomes by type of symptom, suggesting higher rates with increased symptom severity.

### Procedural risks of carotid endarterectomy: asymptomatic patients

In asymptomatic patients, the overall CEA procedural stroke/death risk across 24 studies was 2·00 (95 per cent c.i. 1·49 to 2·58) per cent (*Fig*. 2), with combined procedural risks in studies from 2005 onwards significantly lower than corresponding risks in earlier studies: 1·50 (1·01 to 2·07) *versus* 3·17 (2·39 to 4·06) per cent (*P* < 0·001) (*Fig*. 2). Substantial heterogeneity in rates was observed across individual studies (*Fig. S3*, supporting information). In the meta‐regression analysis, there was a 6·9 (95 per cent c.i. 3·0 to 10·5) per cent per annum reduction in CEA procedural stroke/death rate (*Fig*. 3 *b*). Rates of procedural deaths and strokes, separately, were lower in later studies, but only the reduction in death rate in asymptomatic patients was statistically significant (*Fig*. 2); data on procedural major and minor strokes, separately, are very limited (*Fig. S2*, supporting information). There was no statistically significant difference in procedural MI rates between studies completing recruitment before 2005 and later studies (*P* = 0·434), and the combined rate across all studies was 1·06 (0·77 to 1·40) per cent (*Fig*. 2).

### Procedural risks of carotid artery stenting: symptomatic patients

**Figure 4:**
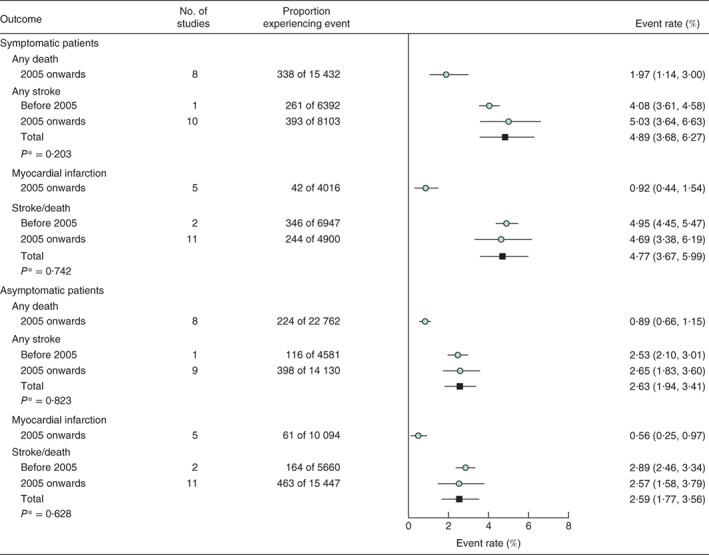
Carotid artery stenting procedural risks in patients classified by symptom status. Adverse event rates are summarized across all studies and separately for those completing recruitment before 2005 or from 2005 onwards. Event rates are shown with 95 per cent confidence intervals. *Before 2005 versus 2005 onwards (2‐sided t test)

**Figure 5:**
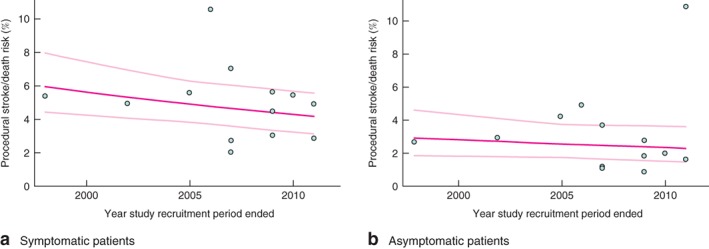
Meta‐regression of carotid artery stenting procedural stroke/death rates among **a** symptomatic and **b** asymptomatic patients. Risks are shown with 95 per cent confidence intervals

The procedural stroke/death risk of CAS in symptomatic patients did not change significantly over time (*P* = 0·742 between studies before 2005 and later studies), with an overall rate across the 13 studies of 4·77 (95 per cent c.i. 3·67 to 5·99) per cent (*Fig*. 4). Substantial heterogeneity in procedural risk was observed across individual studies (*Fig. S4*, supporting information). In the meta‐regression analysis, there was a non‐significant 2·7 (–3·4 to 8·4) per cent per annum reduction in CAS procedural stroke/death rate in symptomatic patients (*P* = 0·374) (*Fig*. 5 *a*). There were no differences between the rates of procedural death and stroke, separately, before 2005 and from 2005 onwards (*Fig*. 4). The ratio between rates of procedural major to minor strokes was about 2 : 3 (1·54 *versus* 2·38 per cent) (*Fig. S5*, supporting information). The combined rate of procedural MI across five contributing studies, all completing recruitment in 2005 or later, was 0·92 (0·44 to 1·54) per cent (*Fig*. 4).

No studies in the review reported procedural outcomes by time since symptoms or by type of symptom.

### Procedural risks of carotid artery stenting: asymptomatic patients

The procedural stroke/death rate of CAS also did not change significantly in asymptomatic patients between studies before 2005 and later studies (*P* = 0·628), with an overall risk of 2·59 (95 per cent c.i. 1·77 to 3·56) per cent across the 13 contributing studies (*Fig*. 4). Substantial heterogeneity in event rates was observed across the individual studies (*Fig. S6*, supporting information). In the meta‐regression analysis, there was a non‐significant 1·8 (–8·1 to 10·9) per cent per annum reduction in procedural CAS stroke/death rate in asymptomatic patients (*P* = 0·704) (*Fig*. 5 *b*). There were no differences in the rates of procedural CAS deaths and strokes, separately, between studies before 2005 and later studies (*Fig*. 4), and the ratio between rates of procedural major and minor strokes was about 2 : 3 (0·87 *versus* 1·46 per cent) (*Fig. S5*, supporting information). The combined rate of procedural MI across the five contributing studies, all completing recruitment from 2005 onwards, was 0·56 (0·25 to 0·97) per cent (*Fig*. 4).

### Sensitivity analyses

The procedural stroke/death risk did not change with the exclusion of studies with a quality score of less than 6 (*Table S4*, supporting information), and there were no clear differences in procedural risks between studies in which outcomes were assessed by an independent neurologist and those in which no assessment was performed by an independent neurologist (*Table S5*, supporting information). In a sensitivity analysis of procedural stroke/death separately for North America and Europe, however, there were some notable differences. Higher procedural CEA stroke/death rates were observed in studies conducted in North America before 2005, with much larger reductions from 2005 onwards among studies conducted in North America compared with those in Europe (*Table S6*, supporting information). The procedural CEA stroke/death rates in studies from 2005 or later were similar in the two regions. The rates of procedural CAS stroke/death were also somewhat higher in North America than in Europe, and remained so in later years (*Table S6*, supporting information).

## Discussion

This systematic review of large observational studies suggests that the procedural risks of CEA, but not CAS, have decreased significantly. Some of the decrease in procedural risks could be due to improvements in medical treatment, better patient selection and increased understanding of the mechanisms of procedural stroke. Since 2003, the use of statins has increased dramatically, and in ACST (Asymptomatic Carotid Surgery Trial) 1^5^ use of lipid‐lowering therapy increased from less than 10 per cent in 1993 to more than 80 per cent when the trial ended in 2007. A subgroup analysis in ACST‐1 suggested that patients on lipid‐lowering therapy had lower perioperative risks^5^. McGirt and colleagues^13^ also reported a lower risk of procedural stroke (1·2 *versus* 4·5 per cent), TIA (1·5 *versus* 3·6 per cent) and mortality (0·3 *versus* 2·1 per cent) with preoperative statin therapy in 1566 patients undergoing CEA^13^. The ACST‐1 data also indicated that a large proportion of procedural CEA strokes are caused by thrombosis or thrombotic occlusion of the ipsilateral carotid artery^34^, with diastolic BP a possible risk factor for procedural stroke/death^35^.

A trend towards centralization of CEA in high‐volume facilities with specialist anaesthetists, neurology and intensive care support, and high‐volume vascular centres and surgeons^36^, may have contributed to the decrease in CEA procedural risks. Furthermore, patients considered high‐risk for CEA are increasingly referred for CAS^12^, which may contribute to the lower risk profile of CEA‐treated patients in cohort studies. The effect of such patient selection, however, is expected to be more limited in large studies such as those included in this systematic review.

There has been less time to establish procedural norms and quality assurance standards for CAS, with the first RCT to include CAS publishing results only in 2004^37^, and operators might have been in an earlier part of the learning curve. With increased operator experience and a better understanding of how best to make use of technological advances in stenting, procedural CAS risks might be expected to fall. For example, it has been suggested that, when operators are more experienced, embolic protection devices (EPDs) result in a lower rate of adverse events^38^. However, although EPDs have been suggested to reduce procedural risks^39^, evidence remains mixed, with distal EPDs shown to cause new ischaemic lesions compared with proximal EPDs^40^.

The procedural risks for symptomatic CEA and CAS in observational studies that completed recruitment in 2005 or later in the present review were lower than those in symptomatic per‐protocol populations in randomized trials: 6·5 per cent for CEA and 7·7 per cent for CAS in the SPACE (Stent‐Protected Angioplasty *versus* Carotid Endarterectomy) trial^41^; 3·9 per cent for CEA and 9·6 per cent for CAS in the EVA‐3S (Endarterectomy *Versus* Angioplasty in Patients with Symptomatic Severe Carotid Stenosis) trial^41^; and 3·4 per cent for CEA and 7·5 per cent for CAS in the ICSS (International Carotid Stenting Study)^42^.

Comparable per‐protocol procedural stroke/death risks data were not available for asymptomatic clinical trial populations. Intention‐to‐treat procedural stroke/death risks in asymptomatic patients in earlier trials were greater than 2·5 per cent for CEA^5^, ^43^, ^44^, but recent trials have reported procedural risks similar to those of observational studies from 2005 onwards reported here: 1·4 per cent for CEA and 2·5 per cent for CAS in CREST (Carotid Revascularization Endarterectomy *versus* Stenting Trial) 1^7^; and 1·7 per cent for CEA and 2·9 per cent for CAS in ACT (Asymptomatic Carotid Trial) 1^9^. In the context of more intensive contemporary medical treatment, procedural risks from 2005 onwards are expected to better represent contemporary practice and contribute to the ongoing debate about the appropriateness of carotid intervention. Procedural risks from 2005 onwards are much lower than current guideline thresholds for acceptable CEA practice (symptomatic 6 per cent, asymptomatic 3 per cent)^45^, but need to be considered in conjunction with a likely lower long‐term stroke risk.

In due course, the pooled results from several completed and ongoing large randomized trials directly comparing CEA with CAS (CREST‐1, ACT‐1, SPACE‐2 and ACST‐2^46^) and CEA/CAS *versus* medical therapy (ECST (European Carotid Surgery Trial) 2^47^ and CREST‐2^48^) will provide reliable contemporary randomized evidence. Until newer RCTs are complete, results from older RCTs remain the standard for comparative analysis between treatment methods. Although observational data cannot provide reliable direct evidence for comparative procedural risks, it can inform absolute risks of particular interventions in categories of patients.

Clinical guidelines and health policy are also informed by the cost‐effectiveness of interventions. However, most cost‐effectiveness studies of CEA and CAS^49^, ^50^, ^51^, ^52^, ^53^, ^54^ have used RCTs or observational cohort studies with patients recruited mostly before 2005. Without recent randomized data, contemporary observational data together with relative risks from randomized trials could inform current clinical practice. In this review, the focus was on procedural risk, but observational data could also inform long‐term stroke risk in patients with carotid stenosis. Recently, calls have been made for the recommendation of medical therapy alone in patients with asymptomatic carotid disease; the proponents claim that, owing to improvements in medical therapy, all asymptomatic patients should be offered medical therapy only and no prophylactic intervention should be considered^55^. However, in guiding such decisions, the stroke/death rates achieved with medical therapy in recent studies should be considered, in view of the decreasing procedural risks of CEA in recent years, rather than the risks in landmark RCTs performed many years ago.

A number of possible limitations of this study should be noted. First, although the inclusion of large observational studies ensured that robust estimates of procedural rates are presented, the lengthy recruitment periods in some of the included studies (60 per cent of studies had recruitment periods longer than 4 years) might have limited the ability of the study to capture changes in procedural risk over time. Second, only limited data were available on procedural MI and major and minor stroke separately, which diminishes the ability to consider the relative importance of different adverse event profiles for CEA and CAS over time. Third, some of the data in the review came from selective carotid registries, which may not have systematically included patients from underperforming centres, instead focusing on better‐performing high‐volume centres. However, this bias is unlikely to have materially influenced the results reported here. Fourth, there were limited data on procedural outcomes by type of symptoms and delay after development of symptoms, which limited discussion of the value of procedure timing with respect to symptoms; reliable evidence on these will be useful to guide recommendations. Finally, a large number of observational cohort studies of patients undergoing CAS, and a smaller proportion of those having CEA, had their outcomes assessed by an independent neurologist. This leads to concerns about a larger number of minor neurological deficits detected in these studies, and under‐reporting of such events in studies where outcomes were not assessed by an independent neurologist. However, no significant differences in procedural stroke/death risks were observed between these types of study, suggesting that the findings are robust to this variation in outcome assessment.

This systematic review, which included nearly 300 000 patients in 51 studies, identified a decrease in procedural stroke/death risk following CEA in both symptomatic and asymptomatic patients in recent years. Procedural risks of CEA were somewhat higher in studies conducted in North America before 2005, and most of the subsequent reduction was also observed in North America; in studies that finished recruiting in 2005 or later, CEA procedural risks were similar in Europe and North America. Procedural risks of CAS appear to have remained stable over time, with a small and not statistically significant decrease in recent years and, again, somewhat higher risks reported in studies conducted in North America. These results suggest that policy guidelines may not reflect contemporary practice and, with ongoing trials still recruiting, recent observational data, with careful allowance for potential biases, may help inform policy.

## Supporting information

Supporting Information
