# Supplementary material for: Meta‐analysis of the procedural risks of carotid endarterectomy and carotid artery stenting over time
Source: Br J Surg. 2017 Dec 4;105(1):26–36. doi: 10.1002/bjs.10717 (PMC5767749; doi:10.1002/bjs.10717)
Supplement: Supplementary file 1 — Supporting Information [file BJS-105-26-s001.docx]

Meta-analysis of the procedural risks of carotid endarterectomy and carotid artery stenting over time

K. Lokuge, D. D. de Waard, A. Halliday, A. Gray, R. Bulbulia and B. Mihaylova

# Appendix S1 Search strategy for observational cohort studies reporting procedural and/or long-term effects of CEA or CAS and number of hits in Medline and Embase

| Search line | Search terms | Medline  to 20^th^ May 2016 | Embase  to 20^th^ May 2016 |
| --- | --- | --- | --- |
| #1 | (Carotid ADJ3 Stenosis) OR (Carotid ADJ2 Artery) | 70733 | 121349 |
| #2 | (Carotid ADJ2 Endarterectom*) OR CEA  OR  (Carotid ADJ2 Stent*) OR CAS OR (Carotid artery angioplasty and stent*) | 87619 | 115939 |
| #3 | peri-procedur* OR peri procedur* OR periprocedur* OR procedur* OR operat* OR peri-operat* OR peri operat* OR perioperat* OR benefit* OR risk* OR complication* OR adverse event* OR adverse effect* OR mortality OR death* OR TIA OR survival OR stroke OR myocard* OR outcom* | 6463904 | 8692914 |
| #4 | registr* OR audit OR database OR cohort OR popul* OR sample OR prospec* | 2848305 | 3795897 |
| #1 AND #2 AND #3 AND #4 (carried forward) | | 2618 | 3924 |

*Note: All searches were performed for Keyword, which incorporates abstract and title*

# Appendix S2 Adapted Newcastle-Ottawa Scale for assessing quality of single-arm observational cohort studies

Note: A study can be awarded a maximum of 8 stars.

Selection:

1) Representativeness of the cohort (max 1 *)

1. truly representative of the carotid stenosis patients in the community (*)
2. somewhat representative of the carotid stenosis patients in the community (*)
3. selected group of users eg nurses, volunteers
4. no description of the derivation of the cohort – i.e no mean age; gender breakdown given

2) [Modified] Clear description of important determinants of procedural and long-term risk in cohort (max 2*)

1. Age, gender, type of symptom (incl degree of stenosis), operator characteristics reported (*)
2. Results presented for important subgroups of participants- by degree of stenosis, age, gender, type of symptom or operator characteristics (*)

3) Ascertainment of exposure: clear ascertainment of procedure performed/ medical therapy

1. secure record (eg surgical records) (*)
2. self-report
3. no description

4) Demonstration that outcome of interest was not present at start of study: the outcome of interest is stroke however it is also the indication for procedure therefor clear description of symptomatic status needed.

1. yes (*)
2. no

Outcome:

1) Assessment of outcome

1. Occurrence of death/stroke/MI well ascertained (record linkage) (*)
2. Occurrence of death/stroke/MI could have been missed (e.g. self-reported data)

2) Was follow-up long enough for outcomes to occur

1. yes (outcome ascertained in all participants at 30 days, follow-up duration of at least 2 years for long-term outcomes) (*)
2. no (e.g. follow-up less than 2 years for long-term outcomes; outcome not ascertained in all participants at 30 days)

3) Adequacy of follow up of cohorts

1. complete follow up - all subjects accounted for(*)
2. subjects lost to follow up unlikely to introduce bias - small number (less than 5%) lost in follow up, or description provided of those lost (*)
3. Greater than 5% loss in follow-up and no description of those lost

# Table S1 Studies excluded due to overlapping study population

| Author | Study setting | Study period | Alternative manuscript used | Study period of alternative manuscript |
| --- | --- | --- | --- | --- |
| De Rango P. (2010)^1^ | Single Center - Uni of Perugia Hospital, Italy (Only Females) | Jan 2004 - Jan 2009 | Parlani G. (2012) | Jan 2001 - March 2009 |
| Dorigo W. (2009)^2^ | Single Centre – Uni of Florence | Jan 1996 – Dec 2007 | Dorigo W. (2011) | Jan 1996 – Dec 2008 |
| Fairman R. (2007)^3^ | CAPTURE | Oct 2004 – Mar 2006 | Gray A. (2007) | Oct 2004 – March 2006 |
| Go C. (2015)^4^ | Single Centre – Division of Vascular Surgery, University of Pittsburgh | Jan 2000 – Dec 2007 | Avgerinos D. (2014) | Jan 2000 – Dec 2008 |
| Gray W. (2011)^5^ | CAPTURE 2 | Mar 2006 – Jan 2009 | Gray A. (2009) and Chaturvedi S. (2010) | Mar 2006 – Jan 2009 |
| Gray W. (2007)^6^ | CAPTURE | Oct 2004 – Mar 2006 | Gray A. (2007) | Oct 2004 – March 2006 |
| Gupta P. 2013^7^ | NSQIP | 2005 - 2010 | Bekelis K. (2013) | 2005 - 2010 |
| Halm E. (2005) ^8^ | Six Hospitals, New York City, USA | Jan 1997 – Dec 1998 | Medicare Beneficiaries of New York – Halm E. (2009) | Jan 1998 – June 1999 |
| Horner R. 2002^9^ | VA centres | Oct 1994 – Sept 1997 | Sidawy A. (2008) | 1996-2003 |
| Jeffrey J. (2011)^10^ | SVS Vascular Registry | Until Oct 2009 | Jeffrey J. (2014) | Until 2011 |
| Jeffrey J. (2012)^11^ | SVS Vascular Registry | Until Dec 2010 | Jeffrey J. (2014) | Until 2011 |
| Kapral M. (2003)^12^ | CEAs performed in Ontario, Canada | 1994 - 1997 | Tu J. (2003) | Jan 1994 – Dec 1997 |
| Kragsterman B. (2004)^13^ | SWEDVASC | Jan 1994 – Dec 1996 | Kragsterman B. (2006) | 1994 - 2003 |
| Kragsterman B. (2006)^14^ | SWEDVASC | 1994 - 2003 | Kragsterman B. (2006) | 1994 - 2003 |
| Matsumura J. (2010) ^15^ | CAPTURE 2 | March 2006 – Jan 2009 | Gray A. (2009) and Chaturvedi S. (2010) | Mar 2006 – Jan 2009 |
| Menyhei G. (2011) ^16^ | VASCUNET | Jan 2003 – Dec 2007 | Paper does not define procedural time period. Study population repeated in Vikatmaa P. (2012) with better definition of procedural time frame. | 2005 - 2010 |
| Pieniążek P. (2012) ^17^ | TARGET-CAS | 2002 - 2010 | Dzierwa K. (2013) | Jan 2001 – Feb 2011 |
| Press M. (2006) ^18^ | Six Hospitals, New York City, USA | Jan 1997 – Dec 1998 | Medicare Beneficiaries of New York – Halm E. (2009) | Jan 1998 – June 1999 |
| Rockman C. (2005)^19^ | New York University Medical Centre | 1992 - 2000 | Rockman C. (2006) | 1992-2003 |
| Schermerhorn M. (2013)^20^ | SVS Vascular Registry | Nov 2000 – Sept 2011 | Jeffrey J. (2014) | Until 2011 |
| Sidawy A. (2009)^21^ | SVS Vascular Registry | Until Dec 2007 | Jeffrey J. (2014) | Until 2011 |
| Stoner M. (2006)^22^ | VA NSQIP | 2000 - 2003 | Sidawy A. (2008) | 1996-2003 |
| Upchurch G. (2014)^23^ | SVS Vascular Registry (limited to patients with contralateral carotid occlusion | Jul 2005 – Dec 2011 | Jeffrey J. (2014) | Until 2011 |
| Wallaert, J. (2012)^24^ | National Surgical Quality Improvement Program (NSQIP) | 2007 - 2009 | Bekelis K. (2013), Garg J. (2011), Wu T. (2015) | 2005-2010; Jan 2005 – Oct 2009; 2005 - 2012 |
| White R. (2010)^25^ | SVS Vascular Registry (only CAS) | Until Nov 2008 | Jeffrey J. (2014) | Until 2011 |
| Wholey, M. (2000)^26^ | Global CAS Registry | 1997 - 1999 | Wholey M. (2003) | June 1997 – Sept 2002 |

# Table S2 Characteristics of observational studies of outcomes of carotid endarterectomy (CEA) or carotid artery stenting (CAS)

| Study and setting | Procedure | Participant characteristics | Recruitment period | Duration of follow-up | Carotid Stenosis characteristics | Symptomatic Definition  Type of symptom | Outcomes reported | Method of outcome assessment |
| --- | --- | --- | --- | --- | --- | --- | --- | --- |
| Assadian A. (2005)^27^  Single Centre - Vienna, Austria | CEA | 1355 CEAs:  58.8% male, 41% asymptomatic, Mean Age: 70.5 years | Jan 1996 – Dec 2002 | 30 days from procedure | Symptomatic patients and asymptomatic patients; degree not stated | Symptomatic – patients have had a prior Stroke or TIA | Procedural:  Death, Stroke | N/A |
| Avgerinos D. (2015)^28^  Single Centre – Division of Vascular Surgery, University of Pittsburgh | CEA | 1342 CEAs:  55.6% male; 64.7% asymptomatic; Mean age: 71.2 | Jan 2000 – Dec 2008 | Mean of 55 months | Symptomatic and asymptomatic; degree of stenosis not stated | Symptomatic – experienced neurological ischemic events such as TIA, amaurosis fugax, or stroke, 6 months before CEA | Procedural:  Death, Stroke, Stroke/Death, MI | Social security death index used to record deaths |
| Ballotta E. (2014)^29^  Single Centre – Padova, Italy | CEA | 2007 CEAs (1773 patients): 70.6% Male; 27.9% Asymptomatic; Mean Age: 75.2 | Jan 1990 – May 2012 | Median: 11.2 years | Symptomatic patients and asymptomatic patients; degree not specified | N/A  N/A | Procedural:  Death, Stroke, Major Stroke, Minor Stroke, Stroke/Death  Long-term:  Death, Stroke, Ipsi Stroke, Contra Stroke (none by Symp status) | By a consultant neurologist |
| Bekelis K. (2013)^30^  NSQIP database, USA | CEA | 35 698CEA patients: 5% Male; 56.1% Asymptomatic; Mean Age: 71.1 | 2005-2010  Follow-up:  30 days from procedure. | 30 days from procedure | Symptomatic and asymptomatic patients; degree not specified | Symptomatic- any subject with a history of cerebrovascular accident with or without neurological deficit or a history of TIA.  63.1% TIA; 35% Stroke | Procedural:  Death, Stroke, MI, Stroke/Death/MI | By a trained nurse |
| Bosiers M. (2007)^31^  AZ St-Blasius, Dendermonde, Belgium;  Imelda Hospital, Bonheiden, Belgium;  University of Sienna, Italy;  Villa Maria Cecilia Hospital,  Cotignola, Italy | CAS | 3179 CAS patients: 58.6% Asymptomatic; Mean Age: 72 | N/A | 30 days from procedure. | Symptomatic and Asymptomatic; degree of stenosis not stated | N/A  N/A | Procedural:  Stroke/Death/TIA | By an independent neurologist |
| Bosiers M. (2005)^32^  ELOCAS Registry – Four high-volume European centres | CAS | 2172 CAS patients (7 technically unsuccessful): 58.4% Asymptomatic; Mean Age: 71 | Feb 1993- Dec 2004 | 5 years | Symptomatic and asymptomatic patients; degree not specified | N/A  N/A | Procedural:  Major Stroke/Death  Long-term:  Stroke/Death | By an independent team of neurologists |
| Calvillo-King L. (2010)^33^  New York Carotid Artery Surgery Study – All Medicare beneficiaries who underwent in New York State, USA | CEA | 6553 CEA patients:  54.9% Male; 100% Asymptomatic; Mean Age: 74.5 | Jan 1998 – June 1999 | 30 days from procedure. | Asymptomatic patients; degree not specified | Asymptomatic patients were defined as those with no history of carotid stroke or TIA <= 12 months before surgery  N/A | Procedural:  Stroke, Death, Stroke/Death | Identified by medical records – major outcomes reviewed by 2 study physicians (including a neurologist) |
| Chaturvedi S. (2010)^34^  CAPTURE 2 – 186 clinical sites in the USA  Conflicts – Abbott Vascular | CAS | 5297 CAS patients:  61.7% Male; 85.3% Asymptomatic; Mean Age: 72.3 | Until Jan 2009  Follow-up:  30 days | 30 days from procedure. | Symptomatic (>=50%) and asymptomatic (>=80%) patients | Symptomatic patients- ipsilateral hemispheric stroke, TIA, and/or amaurosis fugax within the previous 180 days.  N/A | Procedural:  Death, Stroke, MI, Major Stroke, Minor Stroke, Ipsi Stroke, Non-ipsi stroke, Death/Stroke, Death/Stroke/MI, Death/Major Stroke | By an independent neurologist |
| Cremonesi A. (2009)^35^  Villa Maria Cecilia Hospital, Cotignola, Italy | CAS | 1523 CASs (1380 CAS patients) 72.4% Male; 75.9% Asymptomatic; Mean Age: 71.9 | April 1999 – Sept 2007 | 12 months, afterwards through phone interviews (Asymp– 107 months, Symp- 97 months) | Symptomatic (>=50%)and asymptomatic (>=80%) patients | Symptomatic- a lesion related neurological event in the preceding 6 months.  43.7% TIA, 40.7% stroke, 15,5% Amaurosis fugax | Procedural:  Stroke/Death, Major Stroke/ Death, Minor Stroke  Long-term:  30 day stroke/death plus stroke/stroke related death beyond 30 days | Self-reported by patient |
| De Rango P. (2011)^36^  Single Centre study - Perugia, Italy | CAS | 1083 CAS patients: 75.3% Asymptomatic; Mean Age: 71.6 years; 71% Male | Jan 2004 – Mar 2009 | 30 days from procedure. | Symptomatic patients (> 70% stenosis) & asymptomatic patients (> 70% stenosis) by Duplex ultrasound & confirmed at angiography. | Patients were defined symptomatic when ipsilateral hemispheric or retinal symptoms occurred within 6 months from the procedure.  N/A | Procedural:  Stroke/Death | In the presence or suspicion of new neurological or  cardiac events, a team of neurologists and cardiologists was routinely consulted and documented the presence. |
| Dorigo W. (2011)^37^  Single Institution – Uni of Florence, Italy | CEA | 4305 CEAs (3573 patients): 65.8% Asymptomatic; 68.8% Male  Mean Age (Diabetics): 71.3 yrs; Mean Age (Non-Diabetics): 71.7 yrs | Jan 1996 – Dec 2008 | Mean: 40 months | Symptomatic and Asymptomatic; degree of stenosis not stated - NASCET | Patients were considered to be  asymptomatic in the absence of neurological symptoms (TIA or stroke) within 6 months from the intervention  59.2% TIA, 15.5% Stroke, 25.3% Veretrobasilar | Procedural:  Stroke/Death  Long-term:  Death, Stroke (not by Symp status) | By an independent neurologist |
| Dzierwa K. (2013)^38^  Single Centre - Krakow, Poland | CAS | 1252 CASs (1139 patients): 44.6% Asymptomatic; 68.9% Male  Mean Age: 66.3 yrs | Jan 2001 – Feb 2011 | 30 days from procedure. | Symptomatic (>50%) and Asymptomatic (>=80%) - Doppler Duplex ultrasound & computed tomography angiography | Symptomatic- history of TIA, amaurosis fugax or stroke ipsilateral to the ICA stenosis up to 6 months before intervention  N/A | Procedural:  Death, Stroke, Major Stroke, Minor Stroke, Stroke/Death (by addition) | By an independent neurologist |
| Egashira Y. (2014)^39^  Japan using Japanese Registry of Neuroendovascular Therapy | CAS | 5191 CAS:  86.6% male; 40.7% asymptomatic; Mean age: 71.6 | Jan 2008 – Dec 2009 | 30-day follow-up | Symptomatic and asymptomatic; degree of stenosis not stated | N/A  N/A | Procedural:  Stroke | N/A |
| Garg J. (2011)^40^  National Surgical Quality Improvement Program (NSQIP) | CEA | 9285 CEAs:  57.3% Male; Mean Age: Academic Hos – 70.8, Comm Hos – 71.8; | Jan 2005 – Oct 2009 | 30 days from procedure | Symptomatic patients and asymptomatic patients; degree not stated | N/A  N/A | Procedural:  Death, Stroke/Death, Stroke/Death/MI | Data entered by trained clinical nurse reviewers |
| Goode S. (2013)^41^  UK CAS Registry;  31 hospitals in the UK | CAS | 1154 CAS patients: 17% Asymptomatic;  Asymp Male: 74.1%  Symp Male: 67.2% | 1998-2010 | Up to 7 years | Symptomatic and Asymptomatic; <90% of patients have degree of stenosis >50% | N/A  N/A | Procedural:  Death, Stroke/Death, Stroke/Death/MI, Disabling Stroke, Non-disabling stroke, MI  Long-term:  Death | N/A for 30 day outcomes; self-reported for long-term outcome through follow-up form. If form not received then another form sent, and afterwards patients were contacted by telephone. |
| Gray A. (2007)^42^  CAPTURE Registry, 144 sites, United States  Study Sponsor - Abbott Vascular | CAS | 3500 CAS patients: 61.1% Male; 86.2% Asymptomatic; Mean Age: 72.7 | Oct 2004 – March 2006 | 30 days from procedure. | Symptomatic patients (>= 50% stenosis) and asymptomatic patients (>= 80% stenosis) | N/A  N/A | Procedural:  Death, Stroke, Major Stroke, Minor Stroke, MI, Death/Stroke, Death/Major Stroke, Death/Stroke/MI | By two independent neurologists |
| Gray W. (2009)^43^  CAPTURE-2 and EXACT studies  Conflicts – Abbott Vascular | CAS | CAPTURE 2: 4175 CEA patients: 61.7% Male; 86.9% Asymptomatic; Mean Age: 72.5  EXACT 2: 2145 CEA patients:  63.1% Male; 90.1% Asymptomatic; Mean Age: 72.9 | EXACT: Nov 2005-April 2007  CAPTURE 2: Mar 2006 – N/A | 30 days from procedure. | Symptomatic (>=50%) and asymptomatic (>=80%) patients | Symptomatic- experienced TIA, amaurosis fugax, or stroke in the territory supplied by the target vessel within 180 days previous to procedure  N/A | Procedural:  Death/Stroke, Death/Major Stroke | By an independent neurologist |
| Halm E. (2009)^44^  New York Carotid Artery Surgery Study – All Medicare beneficiaries who underwent in New York State, USA | CEA | 9308 CEAs:  55.7% Male; 71.5% Asymptomatic; Mean Age: 74.6 | Jan 1998 – June 1999 | 30 days from procedure. | Symptomatic and asymptomatic patients; degree not specified | Patients without neurological symptoms referable to a carotid artery distribution in the 12 months before surgery were defined as asymptomatic.  66.4% TIA, 27% Minor stroke, 5.42% Major stroke, 13.33% Acute syndromes | Procedural:  Stroke/Death | Identified by medical records – major outcomes reviewed by 2 study physicians (including a neurologist) |
| Hamdan A. (1999)^45^  Beth Israel Deaconess Medical Center, Boston, Massachusetts , USA | CEA | 3092 CEAs (2714 patients): 60.7% Male; 42.6% Asymptomatic; Mean Age: Normal renal func: 70.1  Not-normal renal func: 71.5 | Jan 1990 – Aug 1997 | 30 days from procedure. | Symptomatic and asymptomatic patients; degree not specified | Symptomatic- TIA, episodes of amaurosis fugax, and completed stroke  N/A | Procedural:  Stroke | N/A |
| Hopkins L. (2014)^46^  CABANA study, 99 US centers  Sponsor - Boston Scientific Corporation. | CAS | 1097 CAS patients  67.3% Male; 4% Asymptomatic; Mean Age: 71.3 | Dec 2008 – Oct 2010 | 30 days from procedure. | Symptomatic patients (>= 50% stenosis) and asymptomatic patients (>= 80% stenosis) by ultrasound or angiography (NASTEC) | N/A | Procedural:  Death, Stroke, Major Stroke, Minor Stroke, Ipsi, Contra, MI, Stroke/Death/MI | By an independent neurologist |
| Jalbert J. (2015)^47^  Medicare beneficiaries at least 66 years old | CAS | 22516 CAS patients:  60.5% male, 47.4% asymptomatic, Mean Age: 76.3 years | 2005 - 2009 | Mean of 2 years | Symptomatic and Asymptomatic; degree of stenosis not stated | N/A | Procedural:  Death | Record linkage |
| Jeffrey J. (2014)^48^  SVS Vascular Registry | CEA  CAS | 3373 CAS patients: 1841 Asymptomatic; 60.6% Males ; Mean Age Male:71.2; Female: 71.2 6491 CEA patients: 4038 Asymptomatic; 58.8% Males; Mean Age Male:70.7; Female: 71.0 | 2005 - | 30 days from procedure | Degree of stenosis CEA: 98 (male) ;99% (female)  CAS: 69 (male);74% (female) on average | N/A | Procedural:  Death, Stroke, MI, Death/Stroke/MI. | Analysis based on only patients who had 30 day follow-up visits |
| Kragsterman B. (2006)^49^  Swedvasc, All centres, Sweden | CEA | 6169 CEAs (5808 patients):Mean Age: 70; 66.2% Male; 10.8% Asymptomatic | 1994-2003 | 10 years | Symptomatic and Asymptomatic; degree of stenosis not stated | Asymptomatic stenosis excluded all ipsilateral carotid artery events & non-hemispheric symptoms within 6 months  N/A | Procedural:  Stroke/Death, Death, Fatal Stroke  Long-term:  Death | N/A for procedural outcomes.  Long-term death through record linkage with National Population Registry |
| Kresowik T. (2014)^50^  10 states in the USA (Arkansas, Georgia, Illinois, Indiana, Iowa,  Kentucky, Michigan, Nebraska, Ohio, Oklahoma) | CEA | 19690 CEAs:  40.5% Asymptomatic | 1995-1996  1998-1999 | 30 days from procedure | Symptomatic and Asymptomatic; degree of stenosis not stated | N/A | Procedural:  Death, Stroke/Death | Record linkage and reviewed by 2 independent clinicians |
| Kucey D. (1998)^51^  8 participating centresm Canada | CEA | 1280 CEAs:  27.3% Asymptomatic; 66.1% Male; 23.1% > 75 years | Jan 1994 – Dec 1996 | 30 days from procedure | Symptomatic and Asymptomatic; defined by NASCET criteria | Symptomatic - Ipsilateral hemispheric or ocular event within 120 days of the surgery  40.2% TIA, 5% Moderate Stroke, 34% Minor stroke, 4.15% Amaurosis fugax | Procedural:  Death, Stroke/Death, Non-fatal Stroke | From patient charts |
| Lindstrom D. (2012)^52^  Swedvasc registry | CEA  CAS | 258 CAS patients: 42% Asymptomatic; Mean Age: 69; 74% Male 6474 CEA patients: (79%) symptomatic | Nov 2004 - April 2011 | 30 days from procedure | Symptomatic and asymptomatic patients; degree of stenosis not stated | Symptomatic stenosis was defined as all ipsilateral carotid artery events within 180 days prior to the intervention.  N/A | Procedural:  Stroke/Death | N/A |
| Long W. (2007)^53^  Single Centre – William Beaumont Hospital, Royal Oak, MI, USA | CEA | 1972 CEAs: 59% Male; 63% Asymptomatic; Mean Age: 72.1 | Jan 1999 – Dec 2003 | 30 days from procedure | Symptomatic patients and asymptomatic patients; degree not stated | N/A | Procedural:  Death, Stroke | N/A |
| Lubke T. (2015)^54^  Single Centre - University Hospital of Cologne, Germany | CEA | 1880 CEAs  68.7% Male; 75.7% asymptomatic; Mean Age: 69.9 (Female) 68.7 (Male) | Jan 2000 – Dec 2010 | 11 years | Symptomatic and Asymptomatic; degree of stenosis not stated | N/A | Procedural:  Death, Stroke, Stroke/Death  Long-term:  Death | By the consultant neurologist |
| Lutz. H (2008)^55^  Two teaching hospitals – Giessen and Dessau, Germany | CEA | 1341 CEA patiens: 41.8% Asymptomatic; 73.2% Male; Mean Age GA: 66.5; Mean Age LA: 68.4 | Jan 1995 – Dec 2004  Follow-up:  30 days | 30 days from procedure. | Symptomatic (>70%)and asymptomatic (>80%) patients | Symptomatic – a clear statement of any ipsilateral neurologic deficit in the last six months  52.8% TIA, 38.3% Stroke | Procedural:  Stroke (assumed as post-operative neurological event) | By an independent neurologist |
| Massop D. (2009)^56^  SAPPHIRE Worldwide Registry | CAS | 2001 CAS patients:  72.3% asymptomatic; Mean Age: 72.2; 62% Male | Jan 1989 – Dec 1998 | 30 days from procedure. | Symptomatic (>=50%) and Asymptomatic (>=80%) by ultrasound or angiogram | Symptomatic - TIA or stroke within 180 days of the procedure  N/A | Procedural:  Stroke/Death | By an independent clinical events committee |
| Mattos A. (2001)^57^  Southern Illinois University School of Medicine, USA | CEA | 1249 CEAs (1113 patients): 75.3% Asymptomatic; Mean Age (women): 68.5 years; Age (men):68 years; 61% Male | Mar 1976 – Oct 1997 | Mean (women): 48.6 months  Mean (men): 50.9 months | Symptomatic (> 50% NASCET in 92% women and 95% men) & asymptomatic patients (> 60% ACAS in 99% women and 98% men) | N/A  N/A | Procedural:  Death, Stroke (by Gender subgroup) | Clinic charts, hospital records, referring physicians, telephone conversations with patients |
| Palombo D. (2009)^58^  Italian Vascular Registry – 89 centers in Italy | CEA | 5809 patients (5962 CEAs):  68.7% male, 68.2% asymptomatic, Mean Age: 72.7 years | Jan 2007 – Dec 2007 | 30 days from procedure | Symptomatic patients and asymptomatic patients; degree not stated | N/A  N/A | Procedural:  Stroke | N/A |
| Parlani (2012)^59^  Single Centre - Hospital S. M. Misericordia, Perugia, Italy | CEA  CAS | 2196 procedures: 71% Male; Mean Age: 71.3 years; 31.1% Symptomatic 1116 CEAs;416 Symptomatic; 70.9% Male 1080 CAS; 268 Symptomatic; 71% Male; | Jan 2001 - March 2009 | 6 year follow-up  Mean follow-up: 47.23 months | Symptomatic (>60%) and Asymptomatic (>70%) | Patients were defined as symptomatic when ipsilateral hemispheric or retinal symptoms occurred within 6 months from the procedure  N/A | Procedural:  Stroke/Death  Long-term:  Death, Ischemic Stroke (Not by Symp status) | By an independent neurologist |
| Pulli R. (2005)^60^  Single Centre – Firenze, Italy | CEA | 1883 CEAs (1554 CEA patients):  52.1% Asymptomatic; Mean Age 70 years; 69.9% Male | 1996-2001 | 3 years after procedure | Symptomatic and Asymptomatic; degree of stenosis > 70 in 99.4% of cases | Patients with nonhemispheric symptoms were considered to be asymptomatic  N/A | Procedural:  Stroke/Death | N/A |
| Reed A. (2003)^61^  Brigham & Women’s Hospital, Boston, USA | CEA | 1370 CEAs (1184 patients):  57% Male; 54% Asymptomatic; Mean Age: N/A | Jan 1990 – Dec 1999 | 30 days from procedure. | Symptomatic and asymptomatic patients; degree not specified | N/A  N/A | Procedural:  Death, Stroke , Stroke/Death | N/A |
| Rockman C. (2005)^62^  Six hospitals, New York, USA | CEA | 1972 CEAs:  57.2% Male; 71.3% Asymptomatic; Mean Age: 72.3 | Jan 1997 – Dec 1998 | 30 days from procedure. | Symptomatic and asymptomatic patients; degree not specified  N/A | N/A  N/A | Procedural:  Stroke | Through inpatient results and surgeons post-discharge office records. Two separate and impartial investigators, including a neurologist, independently  reviewed the medical records of all patients |
| Rockman C. (2006)^63^  New York Uni Medical Centre, USA | CEA | 1046 CEAs:  0% Asymptomatic; Mean Age: N/A; 60.3% Male | 1992-2003 | 30 days from procedure | Symptomatic patients only selected; degree of stenosis not stated | Preoperative symptoms were categorized as either hemispheric TIA (including amaurosis fugax) or completed stroke by clinical presentation and the results of brain imaging studies, when available.  Early CEA: 69.9% TIA, 30.1% Stroke  Late CEA: 52.9% TIA, 47.1% Stroke | Procedural:  Death, Stroke, MI | N/A |
| Schreiber T. (2010)^64^  CASES-PMS Study, 73 sites, USA  Study Sponsor- Cordis Corporation | CAS | 1492 CAS patients:  78.2% Symptomatic; Mean Age 73.4 years; 62.7% Male | Aug 2003 – Oct 2005 | 1 year | Symptomatic patients (>= 50% stenosis) and asymptomatic patients (>= 80% stenosis) by ultrasound or angiogram | N/A  N/A | Procedural:  Stroke/Death | By an independent neurologist |
| Šedivý P. (2015)^65^  Single Centre – Na Homolce Hospital, Czech Republic | CEA  CAS | 5363 CEAs:  46.7% Asymptomatic | 1993 – 2014 | 30-day follow-up | Symptomatic and asymptomatic; degree of stenosis not stated | N/A  N/A | Procedural:  Stroke/Death | N/A |
| Setacci C. (2010)^66^  Single Centre study - Sienna, Italy | CAS | 2124 CAS patients: Mean Age: 76.3; 65% Male; 48% Symptomatic | Dec 2000 – May 2009 | 30 days from procedure. | Symptomatic patients (> 70% stenosis) and asymptomatic patients (> 70% stenosis) by Doppler ultrasound | N/A  N/A | Procedural:  Stroke | By an independent neurologist |
| Sidawy A. (2008)^67^  Veterans Affairs National Surgical Quality Improvement Program (VA-NSQIP) – 123 participating medical centres in the USA | CEA | 20899 CEAs (20080 CEA patients): 50.2% Asymptomatic;  Mean Age (GFR >=60, 30-59, M30): 66.7, 71.5, 71.9  Male Age (GFR >=60, 30-59, M30): 98.7%, 98.4%, 98.2%  **GFR: glomerular filtration rate* | Jan 1996 - Dec 2003 | 30 days from procedure in 79% and rest in-hospital | Symptomatic and Asymptomatic; degree of stenosis not stated | Symptomatic - Previous history of Stroke/TIA  N/A | Procedural:  Death  Stroke (a new cerebrovascular accident/ stroke or coma lasting 24 hours), MI (myocardial infarction or cardiac arrest) | Recorded by trained nurse from medical record review, surgeon interview,  or patient follow-up |
| Stabile E. (2010)^68^  Single Centre – Mercogliano, Italy. | CAS | 1300 CAS patients (only those that used PEO for neuroprotection):  71.2% Asymptomatic; Mean Age 71.5 years; 69.9% Male | July 2004 to May 2009 | 30 days from procedure | Symptomatic patients (>= 50% stenosis) and asymptomatic patients (>= 80% stenosis) – NASCET | Symptomatic is defined as carotid stenosis associated, within 6 months before the procedure, with amaurosis fugax, ipsilateral hemispheric TIA, or ipsilateral ischemic stroke not resulting in a major residual neurological deficit  N/A | Procedural:  Stroke/Death | By an independent neurologist |
| Stabile E. (2012)^69^  8 Institutions; Mercogliano, Italy  London, Canada  Cotignola, Italy  Dendermonde, Belgium  Mirano, Italy,  Sienna, Italy  Perugia, Italy  Leipzig, Germany  Frankfurt, Germany | CAS | 1611 CAS patients:  68.6% Male; 72.6% Asymptomatic; Mean Age: 72.1 | Jan 2007 – Dec 2007 | 30 days from procedure | Symptomatic patients (>=50%) and asymptomatic patients (>=80%; life expectancy > 5 years) according to NASCET criteria | Symptomatic - those with ipsilateral amaurosis fugax, ipsilateral hemispheric TIA(s), or ipsilateral ischemic stroke without major disability (Rankin >3) within 6 months before intervention.  N/A | Procedural:  Stroke/Death | Patients assessed after one month with a clinical examination and questionnaire.  An independent neurologist, a cardiologist, and a vascular surgeon reviewed all charts as a part of the auditing process |
| Stromberg S. (2012)^70^  Swedvasc | CEA | 2596 CEA patients:  0% Aymptomatic; Mean Age 71.9 years; 66.7% Male | May 2008 – May 2011 | 30 days from procedure | Symptomatic patients only selected; degree of stenosis not stated | TIA, minor stroke (symptoms restituted within 1 week or remaining minor dysfunction), or major stroke (disabling stroke) 180 days prior to procedure  Procedural stroke/death risks were reported by time for symptom to procedure (0.-2 days: 11.5%; 3-7 days: 3.6%; 8-14 days: 4%; 15-180 days: 5.4%)  40.1% TIA, 35.3% minor stroke, 3% major stroke, 19.6% Amaurosis fugax, 2.1% Crescendo TIA | Procedural:  Death, Stroke, Major Stroke, Minor Stroke, Stroke/Death, | N/A |
| Tu J. (2003)^71^  All CEA’s in Ontario Canada – 34 hospitals | CEA | 6038 CEAs:  65.3% Male; 30.6% Asymptomatic; Mean Age: 68.3 | Jan 1994 – Dec 1997 | 30 days from procedure. | Symptomatic (87% >= 70%) and asymptomatic patients (97% >= 60%) – states *“Most variable definitions were the same as those used in NASCET”* | Patients were considered symptomatic if they had a history of stroke, TIA, or amaurosis fugax within 6 months of surgery.  77.1% TIA or Stroke; 22.8% Amaurosis fugax | Procedural:  Death, Non-fatal Stroke, Stroke/Death | Record linkage |
| UK CEA audit – round 5^72, 73^ | CEA | 16774 CEAs:  67% Male; Mean Age: 72  Data reported for 2150 Asymptomatic patients only | 2011-2013 | 30 days from procedure. | Symptomatic and Asymptomatic; degree of stenosis not stated  Data reported for 2150 Asymptomatic patients only | Symptomatic – History of stroke or TIA  N/A | Procedural:  Death, Stroke, Stroke/Death, MI | Post-hospital discharge follow-up assessment |
| Vikatmaa P. (2012)^74^  VASCUNET - Australia, Denmark, Hungary, Italy, Finland, Norway, Sweden, Switzerland and the United Kingdom | CEA | 53077 CEAs: 39.9% Asymptomatic  Australia (no 30 day outcomes): 33.1% Asymptomatic; Mean Age: 72.4; 70.6% Male  Denmark: 0% Asymptomatic; Mean Age: 67.9; 67.7% Male  Finland: 15.6% Asymptomatic; Mean Age: 68.4; 68.6% Male  Hungary: 46.1% Asymptomatic; Mean Age: 65.9; 61% Male  Italy: 68.6% Asymptomatic; Mean Age: 72.2; 68.2% Male  Norway: 20.5% Asymptomatic; Mean Age: 68.2; 66.9% Male  Sweden: 22.8% Asymptomatic; Mean Age: 70.7; 68% Male  Switzerland: 40.4% Asymptomatic; Mean Age: 70.6; 71.2% Male  UK: 16.8% Asymptomatic; Mean Age: 72.7; 68.9% Male | 2005-09 + UK for 2010 | 30 days from procedure | Symptomatic and Asymptomatic; degree of stenosis not stated | N/A  N/A | Procedural:  Stroke/Death | N/A |
| Wholey M. (2003)^75^  53 participating centres in Europe, South & North America, & Asia – Global CAS Registry | CAS | 12392 CASs (11243 patients):  6392 Symptomatic, 4581 Asymptomatic. | June 1997 – Sept 2002 | 30 days from procedure. | Symptomatic and asymptomatic patients; degree not specified | N/A  N/A | Procedural:  Death (Procedure related), Stroke, Stroke/Death, Minor Stroke, Major Stroke | N/A |
| Wu T. (2015)^76^  National Surgical Quality Improvement Program (NSQIP) | CEA | 24211 CEAs:  58% male, 100% asymptomatic, Mean Age: 65 years | 2005 - 2012 | 30 days from procedure | Asymptomatic; degree of stenosis not stated | Symptomatic – with a previous diagnosis of TIA, stroke, and stroke without neurologic deficit  N/A | Procedural:  Stroke/Death | N/A |
| Yang (2014)^77^  Single Institution - Seol, Korea | CEA  CAS | CEA: 698 CEAs; 253 Symptomatic;87.5% Male CAS:  455 CASs; 234 Symptomatic;85.7% Male; | March1995 - Dec 2012 | 30 days from procedure | Symptomatic and Asymptomatic; degree of stenosis not stated | Presence of neurological or ocular symptoms within 6 months prior to CEA or CAS was regarded as symptomatic  CEA patients: 5.1% Amaurosis fugax, 44.6% TIA, 50.2% stroke  CAS patients: 5.9% Amaurosis fugax, 31.6% TIA, 62.4% stroke | Procedural:  Stroke, Ipsi, Non-Ipsi  by Symp status | N/A |
| Yoshida S. (2013)^78^  Single Centre - Department of Vascular and Endovascular Surgery, Beth Israel Deaconess Medical Centre and Harvard Medical School | CEA  CAS | 271 CAS patients:  66.1% Male; 31.4% Symptomatic; 17.3% > 80 years  830 CEA patients:  56.6% Male; 32.9% Symptomatic; 19.8% > 80 years | Jan 2005 – Dec 2010 | 30 days from procedure | Symptomatic and asymptomatic; degree of stenosis not stated | Patients with nonspecific symptoms or undocumented symptom status were considered asymptomatic for the  purposes of this study | Procedural:  Stroke, Death | N/A |

N/A - Information not available

# Table S3 Quality assessment scores of included studies

| Reference/Study | Adapted single arm Newcastle-Ottawa Quality assessment scale | | | | | | | Total Score |
| --- | --- | --- | --- | --- | --- | --- | --- | --- |
|  | S1 | S2 | S3 | S4 | O1 | O2 | O3 |  |
| Assadian A. (2005) | * | ** | * | * |  | * | * | 7 |
| Avgerinos D. (2014) | * | * | * |  |  | * | * | 5 |
| Ballotta E. (2014) | * | ** | * |  | * | * | * | 7 |
| Bekelis K. (2013) | * | * | * | * | * | * | * | 7 |
| Bosiers M. (2005) | * | ** | * |  | * | * |  | 6 |
| Bosiers M. (2007) | * | * | * |  | * | * | * | 6 |
| Calvillo-King L. (2010) | * | * | * | * | * | * | * | 7 |
| Chaturvedi S. (2010) | * | ** | * | * | * | * | * | 8 |
| Cremonesi A. (2009) | * | ** | * | * |  | * | * | 7 |
| De Rango P. (2011) | * | * | * | * | * | * | * | 7 |
| Dorigo W. (2011) |  | * | * | * | * | * | * | 6 |
| Dzierwa K. (2013) | * | * | * | * | * | * | * | 7 |
| Egashira Y. (2014) |  | * | * | * |  | * | * | 5 |
| Garg J. (2011) | * | * | * | * | * | * | * | 7 |
| Go C. (2015) | * | * | * |  |  | * | * | 5 |
| Goode S. (2013) |  | ** | * |  |  | * | * | 5 |
| Gray A. (2007) | * | ** | * |  | * | * | * | 7 |
| Gray W. (2009) – CAPTURE 2 | * | ** | * | * | * | * | * | 8 |
| Gray W. (2009) - EXACT | * | ** | * | * | * | * | * | 8 |
| Halm E. (2009) | * | * | * | * | * | * | * | 7 |
| Hamdan A. (1999) | * | ** | * | * |  | * | * | 7 |
| Hopkins L. (2014) | * | ** | * |  | * | * |  | 6 |
| Jalbert J. (2015) |  | ** | * | * | * | * | * | 7 |
| Jeffery J. (2014) |  | ** | * | * |  | * | * | 6 |
| Kragsterman B. (2006) | * | * | * | * |  | * | * | 6 |
| Kresowik T. (2014) |  | * | * |  | * | * | * | 5 |
| Kucey D. (1998) | * | ** | * | * | * | * | * | 8 |
| Lindstrom D. (2012) | * | * | * | * |  | * | * | 7 |
| Long W. (2007) | * | * | * | * |  | * | * | 6 |
| Lübke T. (2015) | * | ** | * |  | * | * | * | 7 |
| Lutz. H (2008) | * | ** | * | * | * | * | * | 8 |
| Massop D. (2009) | * | * | * | * | * | * | * | 7 |
| Mattos A. (2001) | * | ** | * |  |  | * | * | 6 |
| Palombo D. (2009) | * | * | * | * | * | * | * | 7 |
| Parlani (2012) | * | * | * | * | * | * | * | 7 |
| Pulli R. (2005) | * | ** | * | * |  | * | * | 7 |
| Reed A. (2003) | * | ** | * |  |  | * | * | 6 |
| Rockman C. (2005) | * | ** | * |  | * | * | * | 7 |
| Rockman C. (2006) | * | * | * | * |  | * | * | 6 |
| Schreiber T. (2010) | * | ** | * |  | * | * | * | 7 |
| Šedivý P. (2015) | * | * | * |  |  | * | * | 5 |
| Setacci C. (2010) |  | * | * |  | * | * | * | 5 |
| Sidawy A. (2008) | * | * | * | * | * | * | * | 7 |
| Stabile E. (2010) |  | ** | * | * | * | * | * | 7 |
| Stabile E. (2012) | * | * | * | * | * | * | * | 7 |
| Stromberg S. (2012) | * | * | * | * |  | * | * | 6 |
| Tu J. (2003) | * | ** | * | * | * | * | * | 8 |
| UK CEA audit – round 5 | * | * | * | * | * | * | * | 7 |
| Vikatmaa P. (2012) | * | ** | * |  |  | * | * | 6 |
| Wholey M. (2003) | * | * | * |  |  | * |  | 4 |
| Wu T. (2015) | * | ** | * | * |  | * | * | 7 |
| Yang (2014) | * | ** | * | * | * | * | * | 7 |
| Yoshida S. (2013) | * | ** | * | * |  |  | * | 5 |

# Table S4 Meta-analysis of procedural Stroke/Death rates restricted to studies with quality score ≥6

| Procedure | Patient Symptomatic Status | End of Study period | # Studies | Events | No. at risk | Procedural Stroke/Death Rate  (95% CI) | p-value for difference between pre 2005 and post 2005 rates¹ |
| --- | --- | --- | --- | --- | --- | --- | --- |
| CEA | Symptomatic | Pre-2005 | 6 | 766 | 14485 | 4.46% (2.65%, 6.70%) | 0.031 |
|  |  | Post-2005 | 13 | 928 | 39290 | 2.52% (1.97%, 3.13%) |  |
| CEA | Asymptomatic | Pre-2005 | 6 | 348 | 11203 | 2.88% (1.93%, 4.01%) | 0.021 |
|  |  | Post-2005 | 14 | 689 | 51772 | 1.50% (0.96%, 2.15%) |  |
| CAS | Symptomatic | Pre-2005 | 1 | 30 | 555 | 5.41% (3.67%, 7.45%) | 0.524 |
|  |  | Post-2005 | 10 | 192 | 3947 | 4.61% (3.18%, 6.28%) |  |
| CAS | Asymptomatic | Pre-2005 | 1 | 29 | 1079 | 2.69% (1.80%, 3.74%) | 0.952 |
|  |  | Post-2005 | 10 | 459 | 15246 | 2.64% (1.54%, 4.00%) |  |
| ¹p-values from t-tests for differences of procedural stroke/death risks between pre- and post-2005 studies. | | | | | | | |

# Table S5 Procedural stroke/death rates of CEA and CAS in studies classified by method of outcome assessment

| Procedure | Patient Symptomatic Status | Method of outcome assessment | End of Study recruitment | Number of Studies | Number Procedural Stroke/Death events | Number of patients | Procedural Stroke/Death Rate  (95% CI) | p-values for difference in rates: | | |
| --- | --- | --- | --- | --- | --- | --- | --- | --- | --- | --- |
|  |  |  |  |  |  |  |  | between pre-and post-2005 | Pre-2005: between studies assessed by independent neurologist or not | Post-2005: between studies assessed by independent neurologist or not |
| CEA | Symptomatic | By independent neurologist | Pre-2005 | 0 |  |  |  | N/A | N/A | 0.695 |
|  |  |  | Post-2005 | 2 | 35 | 1886 | 2.04% (0.93%, 3.54%) |  |  |  |
|  |  | Not by an independent neurologist | Pre-2005 | 2 | 444 | 6847 | 6.48% (5.91%, 7.08%) | <0.001 |  |  |
|  |  |  | Post-2005 | 3 | 221 | 9120 | 2.40% (1.44%. 3.58%) |  |  |  |
|  |  |  | All Studies | 5 | 665 | 15967 | 3.89% (2.10%, 6.21%) |  |  |  |
| CEA | Asymptomatic | By independent neurologist | Pre-2005 | 0 |  |  |  | N/A | N/A | 0.806 |
|  |  |  | Post-2005 | 2 | 38 | 3535 | 1.14% (0.61%, 1.82%) |  |  |  |
|  |  | Not by an independent neurologist | Pre-2005 | 4 | 603 | 16483 | 3.83% (3.16%, 4.56%) | 0.27 |  |  |
|  |  |  | Post-2005 | 3 | 33 | 3738 | 0.80% (0.01%, 2.59%) |  |  |  |
|  |  |  | All Studies | 7 | 636 | 20221 | 2.32% (1.03%, 4.10%) |  |  |  |
| CAS | Symptomatic | By independent neurologist | Pre-2005 | 1 | 30 | 555 | 5.41% (3.67%, 7.45%) | 0.737 | N/A | 0.309 |
|  |  |  | Post-2005 | 7 | 163 | 3171 | 4.91% (2.99%, 7.25%) |  |  |  |
|  |  |  | All Studies | 8 | 193 | 3726 | 4.96% (3.27%, 6.98%) |  |  |  |
|  |  | Not by an independent neurologist | Pre-2005 | 0 |  |  |  | N/A |  |  |
|  |  |  | Post-2005 | 2 | 22 | 634 | 3.45% (1.93%, 5.37%) |  |  |  |
| CAS | Asymptomatic | By independent neurologist | Pre-2005 | 1 | 29 | 1079 | 2.69% (1.80%, 3.74%) | 0.879 | N/A | 0.087 |
|  |  |  | Post-2005 | 7 | 419 | 13173 | 2.57% (1.51%, 3.89%) |  |  |  |
|  |  |  | All Studies | 8 | 448 | 14252 | 2.59% (1.65%, 3.72%) |  |  |  |
|  |  | Not by an independent neurologist | Pre-2005 | 0 |  |  |  | N/A |  |  |
|  |  |  | Post-2005 | 2 | 29 | 1972 | 1.47% (0.91%, 2.15%) |  |  |  |

# Table S6 Procedural Stroke/Death risks of CEA and CAS separately in studies conducted in North America and Europe

| Procedure | Patient Symptomatic Status | Study region | End of Study recruitment | Number of Studies | Number Procedural Stroke/Death events | Number of patients | Procedural Stroke/Death Rate  (95% CI) | p-values for difference in rates: | | |
| --- | --- | --- | --- | --- | --- | --- | --- | --- | --- | --- |
|  |  |  |  |  |  |  |  | between pre-and post-2005 | Pre-2005: between North America and Europe | Post- 2005:  between North America and Europe |
| CEA | Symptomatic | North America | Pre-2005 | 6 | 833 | 12597 | 6.09% (4.52%, 7.86%) | 0.072 | 0.198 | 0.576 |
|  |  |  | Post-2005 | 2 | 217 | 8577 | 3.19% (1.50%, 5.48%) |  |  |  |
|  |  |  | All | 8 | 1050 | 21174 | 5.30% (3.80%, 7.04%) |  |  |  |
|  |  | Europe | Pre-2005 | 2 | 241 | 6081 | 2.78% (0.62%, 6.37%) | 0.924 |  |  |
|  |  |  | Post-2005 | 14 | 906 | 36639 | 2.62% (2.02%, 3.30%) |  |  |  |
|  |  |  | All | 16 | 1147 | 42720 | 2.65% (2.07%, 3.30%) |  |  |  |
| CEA | Asymptomatic | North America | Pre-2005 | 6 | 638 | 17577 | 3.70% (3.13%, 4.32%) | <0.001 | 0.013 | 0.595 |
|  |  |  | Post-2005 | 2 | 387 | 25079 | 1.70% (1.13%, 2.38%) |  |  |  |
|  |  |  | All | 8 | 1025 | 42656 | 3.15% (2.37%, 4.03%) |  |  |  |
|  |  | Europe | Pre-2005 | 2 | 26 | 1610 | 1.64% (0.80%, 2.76%) | 0.762 |  |  |
|  |  |  | Post-2005 | 14 | 346 | 30068 | 1.46% (0.92%, 2.11%) |  |  |  |
|  |  |  | All | 16 | 372 | 31678 | 1.48% (0.99%, 2.05%) |  |  |  |
| CAS | Symptomatic | North America | Pre-2005 | 1 | 30 | 555 | 5.41% (3.67%, 7.45%) | 0.267 | N/A | <0.001 |
|  |  |  | Post-2005 | 5 | 125 | 1738 | 7.12% (4.95%, 9.63%) |  |  |  |
|  |  |  | All | 6 | 155 | 2293 | 6.72% (4.95%, 8.73%) |  |  |  |
|  |  | Europe | Pre-2005 | 0 |  |  |  | N/A |  |  |
|  |  |  | Post-2005 | 7 | 119 | 3162 | 3.49% (2.53%, 4.60%) |  |  |  |
| CAS | Asymptomatic | North America | Pre-2005 | 1 | 29 | 1079 | 2.69% (1.80%, 3.74%) | 0.12 | N/A | 0.076 |
|  |  |  | Post-2005 | 4 | 388 | 10443 | 3.83% (2.92%, 4.85%) |  |  |  |
|  |  |  | All | 5 | 417 | 11522 | 3.61% (2.81%, 4.51%) |  |  |  |
|  |  | Europe | Pre-2005 | 0 |  |  |  | N/A |  |  |
|  |  |  | Post-2005 | 7 | 75 | 5004 | 1.89% (0.77%, 3.45%) |  |  |  |

Adverse event rates summarised separately for studies completing recruitment before year 2005 (“Pre-2005”) or thereafter (“Post-2005”). North America: 12 USA, 3 Canada; Europe: 9 Italy, 5 Sweden, 3 UK, 2 Germany, 1 Belgium, 1 Czech Republic, 1 Poland, 1 Denmark, 1 Finland, 1 Hungary, 1 Norway, 1 Switzerland

# Figure S1 CEA procedural stroke/death rates in symptomatic patients, by study


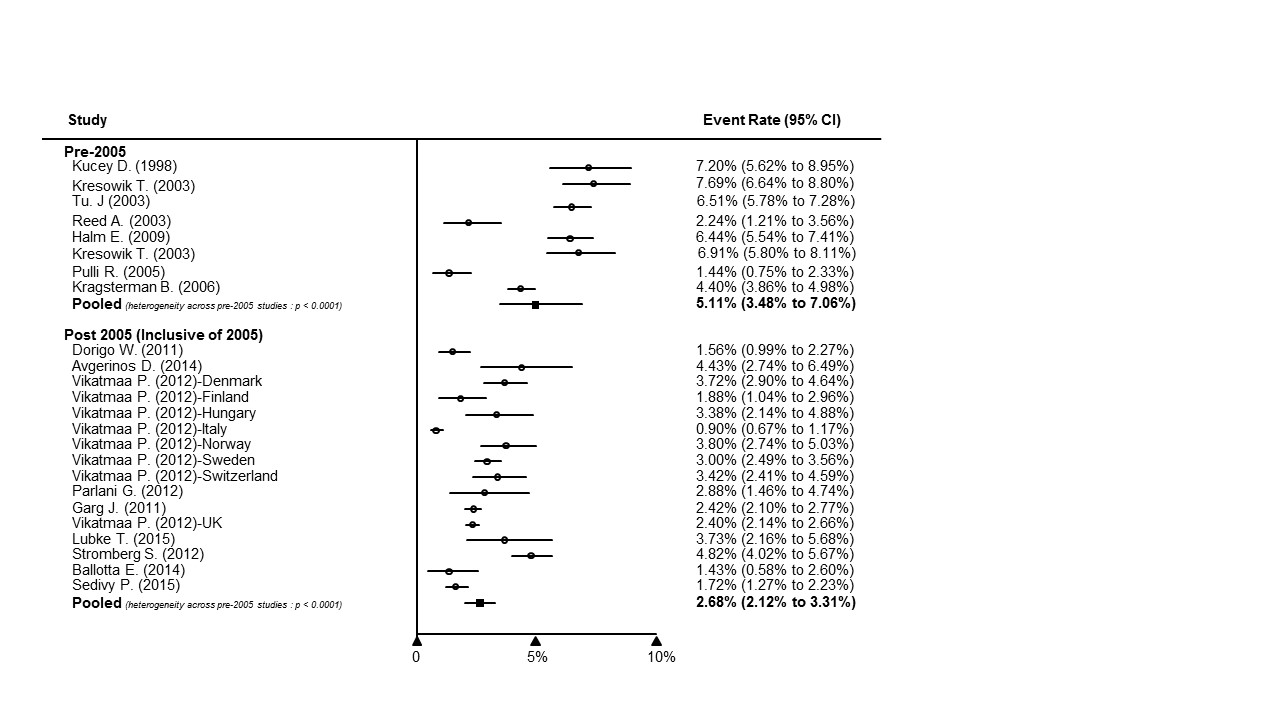


Note: Studies ordered by end of study recruitment year.

Figure S2 Other procedural outcomes of CEA


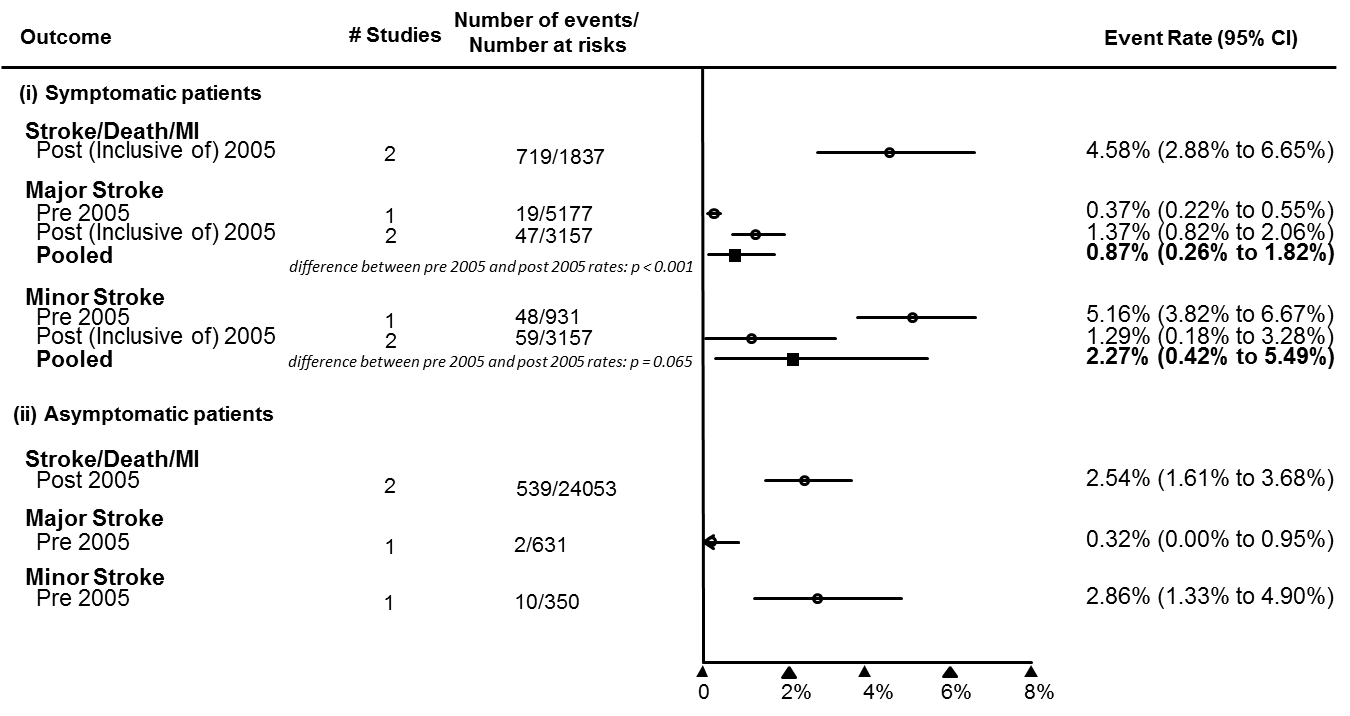


CEA, coronary endarterectomy; MI, myocardial infarction.

Minor stroke defined as modified Rankin Scale (mRS) <3; Major stroke defined as mRS >=3.

Figure S3 CEA procedural stroke/death rates in asymptomatic patients, by study


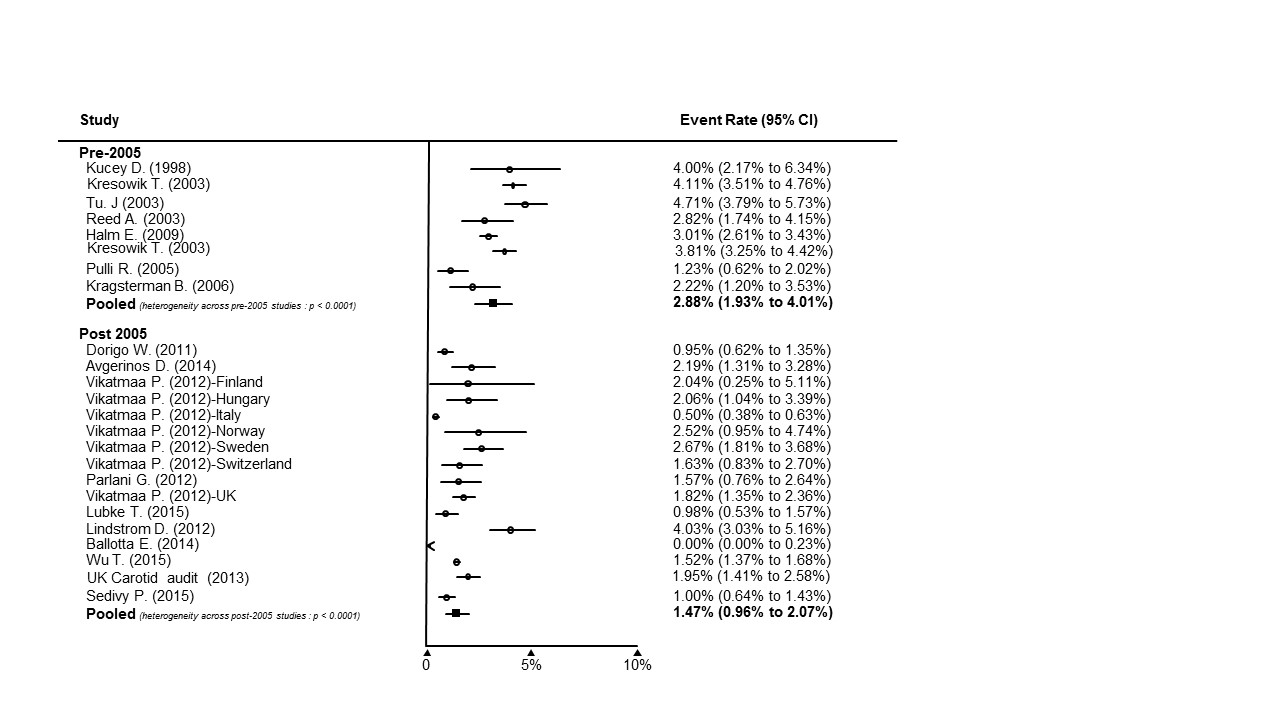


Note: Studies ordered by the end of recruitment year.

# Figure S4 CAS procedural stroke/death rates in symptomatic patients, by study


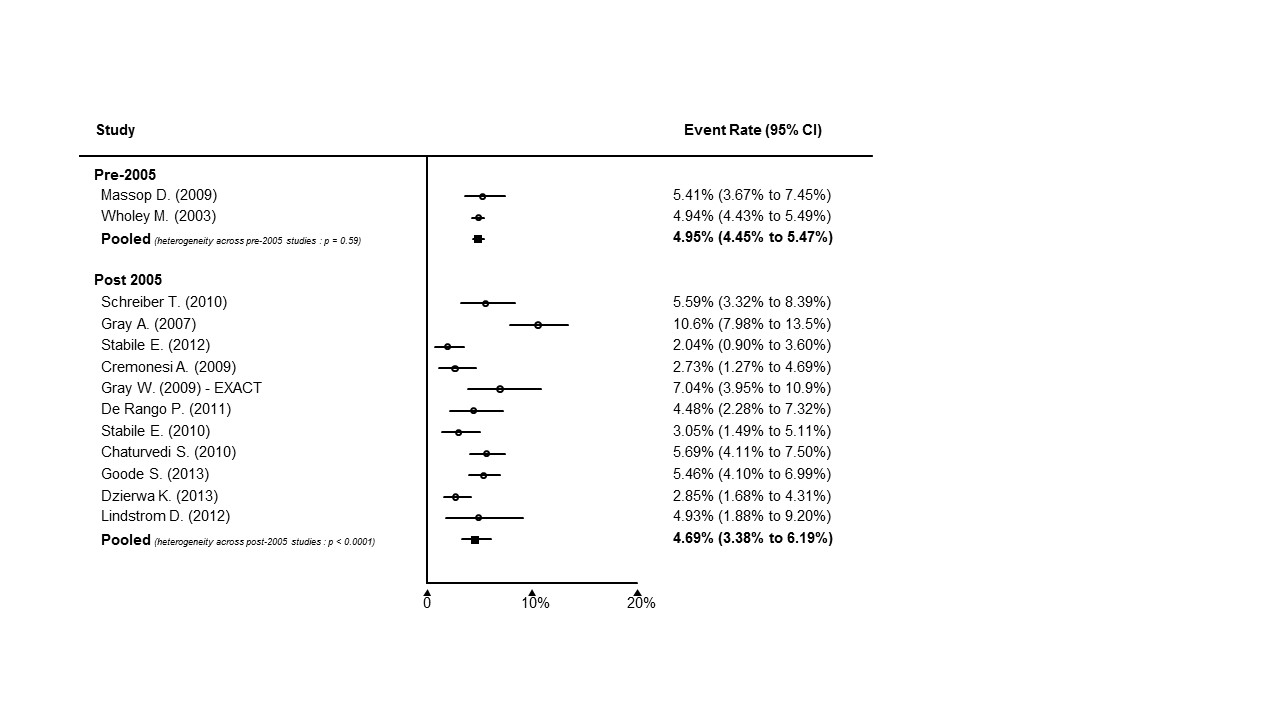


Note: Studies ordered by end of study recruitment year.

# Figure S5 Other procedural outcomes of CAS


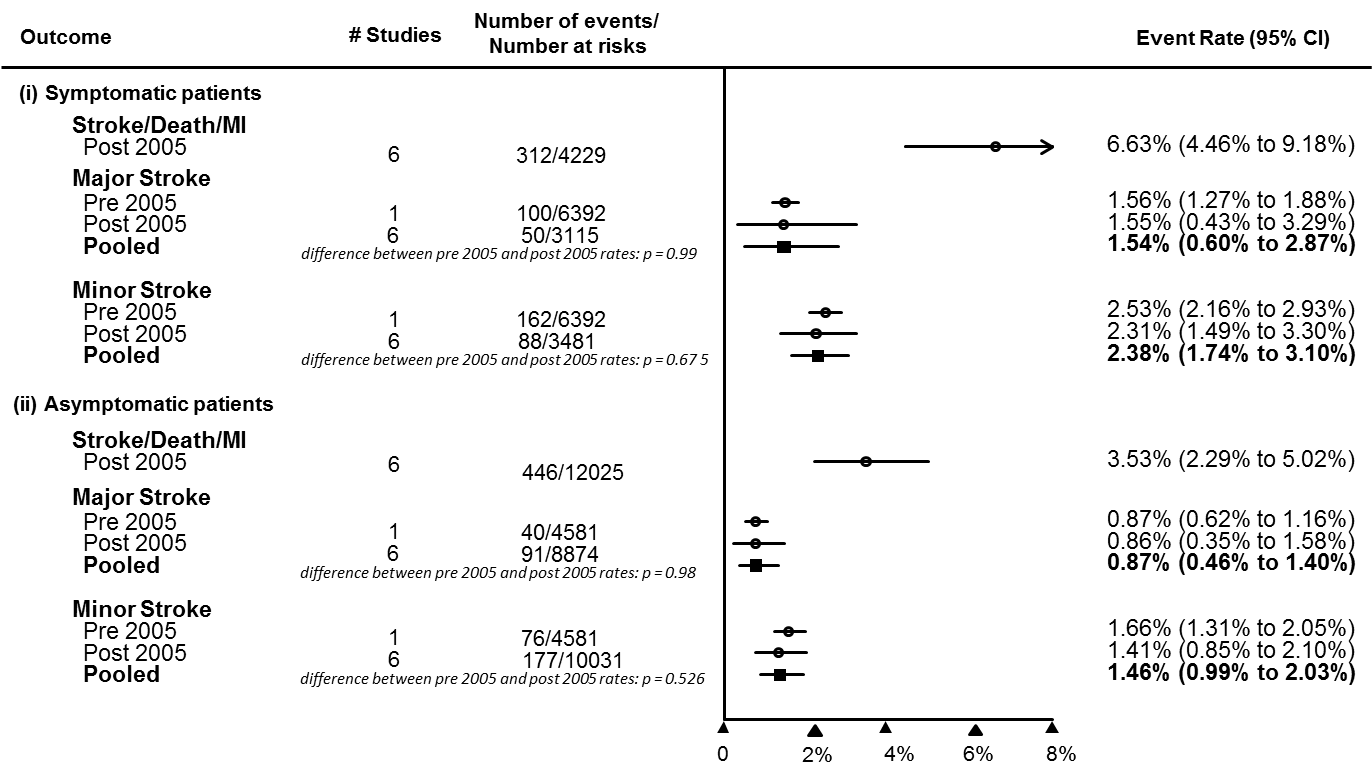


CAS, carotid artery stenting; MI, myocardial infarction.

Minor stroke defined as modified Rankin Scale (mRS) <3; Major stroke defined as mRS ≥3.

# Figure S6 CAS procedural stroke/death rates in asymptomatic patients, by study


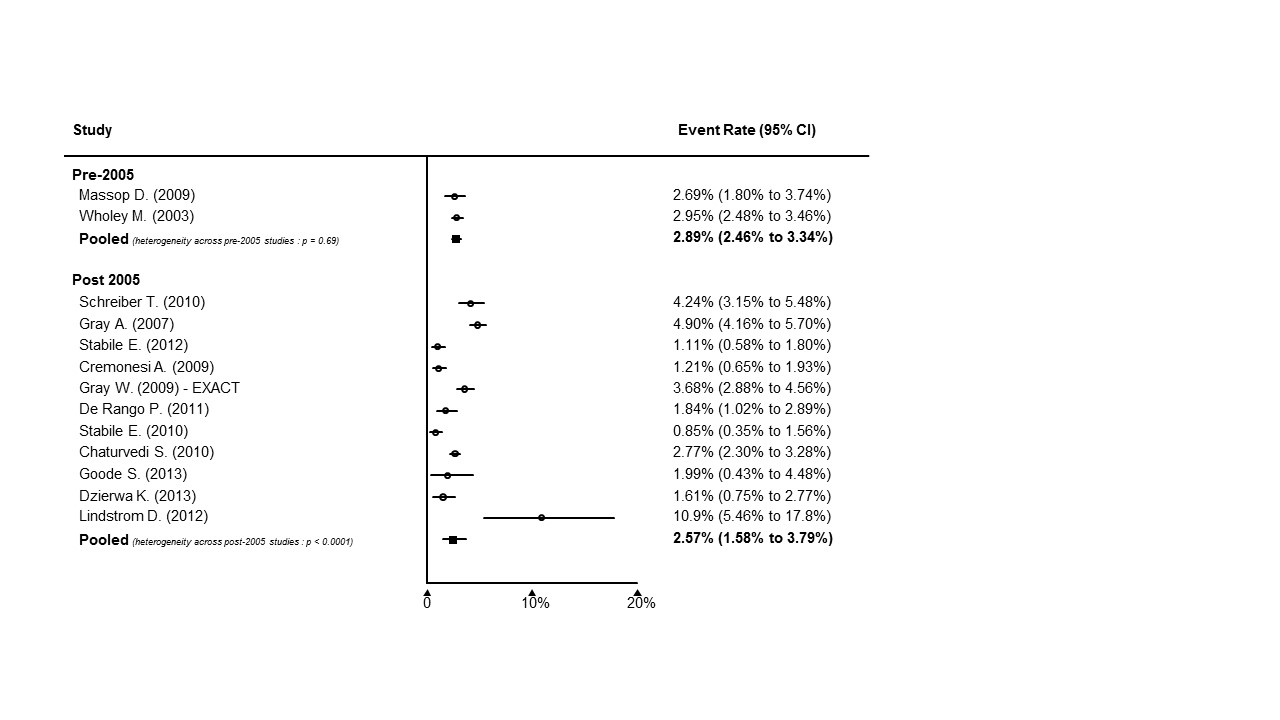


Note: Studies ordered by year recruitment completed in study.

Appendix S3 References

1. De Rango P, Parlani G, Caso V, Verzini F, Giordano G, Cieri E, et al. A comparative analysis of the outcomes of carotid stenting and carotid endarterectomy in women. Journal of Vascular Surgery. 2010; 51(2): 337-44.

2. Dorigo W, Pulli R, Marek J, Troisi N, Pratesi G, Innocenti AA, et al. Carotid endarterectomy in female patients. Journal of Vascular Surgery. 2009; 50(6): 1301-7.

3. Fairman R, Gray WA, Scicli AP, Wilburn O, Verta P, Atkinson R, et al. The CAPTURE registry - Analysis of strokes resulting from carotid artery stenting in the post approval setting: Timing, location, severity, and type. Annals of Surgery. 2007; 246(4): 551-6.

4. Go C, Avgerinos ED, Chaer RA, Ling J, Wazen J, Marone L, et al. Long-term clinical outcomes and cardiovascular events after carotid endarterectomy. Ann Vasc Surg. 2015; 29(6): 1265-71.

5. Mahoney EM, Greenberg D, Lavelle TA, Natarajan A, Berezin R, Ishak KJ, et al. Costs and cost‐effectiveness of carotid stenting versus endarterectomy for patients at increased surgical risk: Results from the SAPPHIRE trial. Catheterization and Cardiovascular Interventions. 2011; 77(4): 463-72.

6. Gray WA, Yadav JS, Verta P, Scicli A, Fairman R, Wholey M, et al. The CAPTURE Registry: Results of carotid stenting with embolic protection in the post approval setting. Catheterization and Cardiovascular Interventions. 2007; 69(3): 341-8.

7. Gupta PK, Ramanan B, MacTaggart JN, Sundaram A, Fang X, Gupta H, et al. Risk index for predicting perioperative stroke, myocardial infarction, or death risk in asymptomatic patients undergoing carotid endarterectomy. Journal of vascular surgery. 2013; 57(2): 318-26.

8. Halm EA, Hannan EL, Rojas M, Tuhrim S, Riles TS, Rockman CB, et al. Clinical and operative predictors of outcomes of carotid endarterectomy. Journal of Vascular Surgery. 2005; 42(3): 420-8.

9. Horner RD, Oddone EZ, Stechuchak KM, Grambow SC, Gray J, Khuri SF, et al. Racial variations in postoperative outcomes of carotid endarterectomy: evidence from the Veterans Affairs National Surgical Quality Improvement Program. Medical care. 2002; 40(1): I-35-I-43.

10. Jim J, Rubin BG, Landis GS, Kenwood CT, Siami FS, Sicard GA, et al. Society for Vascular Surgery Vascular Registry evaluation of stent cell design on carotid artery stenting outcomes. Journal of Vascular Surgery. 2011; 54(1): 71-9.

11. Jim J, Rubin BG, Ricotta JJ, 2nd, Kenwood CT, Siami FS, Sicard GA, et al. Society for Vascular Surgery (SVS) Vascular Registry evaluation of comparative effectiveness of carotid revascularization procedures stratified by Medicare age. Journal of Vascular Surgery. 2012; 55(5): 1313-20; discussion 21.

12. Kapral MK, Wang H, Austin PC, Fang J, Kucey D, Bowyer B, et al. Sex differences in carotid endarterectomy outcomes: results from the Ontario Carotid Endarterectomy Registry. Stroke. 2003; 34(5): 1120-5.

13. Kragsterman B, Logason K, Ahari A, TroengTroeng T, Parsson H, Bergqvist D. Risk factors for complications after carotid endarterectomy - A population-based study. European Journal of Vascular and Endovascular Surgery. 2004; 28(1): 98-103.

14. Kragsterman B, Parsson H, Lindback J, Bergqvist D, Bjorck M, Swedish Vascular R. Outcomes of carotid endarterectomy for asymptomatic stenosis in Sweden are improving: Results from a population-based registry. Journal of Vascular Surgery. 2006; 44(1): 79-85.

15. Matsumura JS, Gray W, Chaturvedi S, Gao X, Cheng J, Verta P, et al. CAPTURE 2 risk-adjusted stroke outcome benchmarks for carotid artery stenting with distal embolic protection. Journal of Vascular Surgery. 2010; 52(3): 576-83, 83.e1-83.e2.

16. Menyhei G, Bjorck M, Beiles B, Halbakken E, Jensen LP, Lees T, et al. Outcome following carotid endarterectomy: Lessons learned from a large international vascular registry. European Journal of Vascular and Endovascular Surgery. 2011; 41(6): 735-40.

17. Pieniazek P, Tekieli L, Musialek P, Kablak Ziembicka A, Przewlocki T, Motyl R, et al. Carotid artery stenting according to the tailored-CAS algorithm is associated with a low complication rate at 30 days: data from the TARGET-CAS study. Kardiol Pol. 2012; 70(4): 378-86.

18. Press MJ, Chassin MR, Wang J, Tuhrim S, Halm EA. Predicting medical and surgical complications of carotid endarterectomy: comparing the risk indexes. Arch Intern Med. 2006; 166(8): 914-20.

19. Rockman CB, Saltzberg SS, Maldonado TS, Adelman MA, Cayne NS, Lamparello PJ, et al. The safety of carotid endarterectomy in diabetic patients: clinical predictors of adverse outcome. Journal of Vascular Surgery. 2005; 42(5): 878-83.

20. Schermerhorn ML, Fokkema M, Goodney P, Dillavou ED, Jim J, Kenwood CT, et al. The impact of Centers for Medicare and Medicaid Services high-risk criteria on outcome after carotid endarterectomy and carotid artery stenting in the SVS Vascular Registry. Journal of Vascular Surgery. 2013; 57(5): 1318-24.

21. Sidawy AN, Zwolak RM, White RA, Siami FS, Schermerhorn ML, Sicard GA, et al. Risk-adjusted 30-day outcomes of carotid stenting and endarterectomy: results from the SVS Vascular Registry. Journal of Vascular Surgery. 2009; 49(1): 71-9.

22. Stoner MC, Abbott WM, Wong DR, Hua HT, LaMuraglia GM, Kwolek CJ, et al. Defining the high-risk patient for carotid endarterectomy: an analysis of the prospective National Surgical Quality Improvement Program database. Journal of vascular surgery. 2006; 43(2): 285-96. e2.

23. Upchurch GR, Landis GS, Kenwood CT, Siami FS, Tsilimparis N, Ricotta JJ, et al. The influence of contralateral occlusion on results of carotid interventions from the Society for Vascular Surgery Vascular Registry. Journal of Vascular Surgery. 2014; 60(4): 958-65.e2.

24. Wallaert JB, De Martino RR, Finlayson SR, Walsh DB, Corriere MA, Stone DH, et al. Carotid endarterectomy in asymptomatic patients with limited life expectancy. Stroke. 2012; 43(7): 1781-7.

25. White RA, Sicard GA, Zwolak RM, Sidawy AN, Schermerhorn ML, Shackelton RJ, et al. Society of vascular surgery vascular registry comparison of carotid artery stenting outcomes for atherosclerotic vs nonatherosclerotic carotid artery disease. Journal of Vascular Surgery. 2010; 51(5): 1116-23.

26. Wholey MH, Wholey M, Mathias K, Roubin GS, Diethrich EB, Henry M, et al. Global experience in cervical carotid artery stent placement. Catheter Cardiovasc Interv. 2000; 50(2): 160-7.

27. Assadian A, Senekowitsch C, Assadian O, Ptakovsky H, Hagmuller GW. Perioperative morbidity and mortality of carotid artery surgery under loco-regional anaesthesia. Vasa - Journal of Vascular Diseases. 2005; 34(1): 41-5.

28. Avgerinos ED, Go C, Ling J, Makaroun MS, Chaer RA. Survival and long-term cardiovascular outcomes after carotid endarterectomy in patients with chronic renal insufficiency. Ann Vasc Surg. 2015; 29(1): 15-21.

29. Ballotta E, Toniato A, Da Giau G, Lorenzetti R, Da Roit A, Baracchini C. Durability of eversion carotid endarterectomy. Journal of Vascular Surgery. 2014; 59(5): 1274-81.

30. Bekelis K, Bakhoum SF, Desai A, Mackenzie TA, Goodney P, Labropoulos N. A risk factor-based predictive model of outcomes in carotid endarterectomy: The national surgical quality improvement program 2005-2010. Stroke. 2013; 44(4): 1085-90.

31. Bosiers M, de Donato G, Deloose K, Verbist J, Peeters P, Castriota F, et al. Does Free Cell Area Influence the Outcome in Carotid Artery Stenting? European Journal of Vascular and Endovascular Surgery. 2007; 33(2): 135-41.

32. Bosiers M, Peeters P, Deloose K, Verbist J, Sievert H, Sugita J, et al. Does carotid artery stenting work on the long run: 5-year results in high-volume centers (ELOCAS Registry). Journal of Cardiovascular Surgery. 2005; 46(3): 241-7.

33. Calvillo-King L, Xuan L, Zhang S, Tuhrim S, Halm EA. Predicting risk of perioperative death and stroke after carotid endarterectomy in asymptomatic patients: derivation and validation of a clinical risk score. Stroke. 2010; 41(12): 2786-94.

34. Chaturvedi S, Matsumura JS, Gray W, Xu C, Verta P, Investigators C, et al. Carotid artery stenting in octogenarians: periprocedural stroke risk predictor analysis from the multicenter Carotid ACCULINK/ACCUNET Post Approval Trial to Uncover Rare Events (CAPTURE 2) clinical trial. Stroke. 2010; 41(4): 757-64.

35. Cremonesi A, Gieowarsingh S, Spagnolo B, Manetti R, Liso A, Furgieri A, et al. Safety, efficacy and long-term durability of endovascular therapy for carotid artery disease: The tailored-Carotid Artery Stenting Experience of a single high-volume centre (tailored-CASE Registry). EuroIntervention. 2009; 5(5): 589-98.

36. De Rango P, Parlani G, Romano L, Verzini F, Giordano G, Cieri E, et al. Second-generation thienopyridine use is not associated with better early perioperative outcome during carotid stenting. European Journal of Vascular and Endovascular Surgery. 2011; 41(2): 214-21.

37. Dorigo W, Pulli R, Pratesi G, Fargion A, Marek J, Innocenti AA, et al. Early and long-term results of carotid endarterectomy in diabetic patients. Journal of Vascular Surgery. 2011; 53(1): 44-52.

38. Dzierwa K, Pieniazek P, Tekieli L, Musialek P, Przewlocki T, Kablak-Ziembicka A, et al. Carotid artery stenting according to the "tailored CAS" algorithm performed in the very elderly patients: The thirty day outcome. Catheterization and Cardiovascular Interventions. 2013; 82(5): 681-8.

39. Egashira Y, Yoshimura S, Sakai N, Enomoto Y. Real-world experience of carotid artery stenting in Japan: Analysis of 7,134 cases from JR-NET1 and 2 nationwide retrospective multi-center registries. Neurologia Medico-Chirurgica. 2014; 54(1): 32-9.

40. Garg J, Frankel DA, Dilley RB. Carotid endarterectomy in academic versus community hospitals: the national surgical quality improvement program data. Ann Vasc Surg. 2011; 25(4): 433-41.

41. Goode SD, Cleveland TJ, Gaines PA. United Kingdom carotid artery stent registry: Short- and long-term outcomes. CardioVascular and Interventional Radiology. 2013; 36(5): 1221-31.

42. Gray WA, Yadav JS, Verta P, Scicli A, Fairman R, Wholey M, et al. The CAPTURE registry: predictors of outcomes in carotid artery stenting with embolic protection for high surgical risk patients in the early post-approval setting. Catheter Cardiovasc Interv. 2007; 70(7): 1025-33.

43. Gray WA, Chaturvedi S, Verta P, Investigators, the Executive C. Thirty-day outcomes for carotid artery stenting in 6320 patients from 2 prospective, multicenter, high-surgical-risk registries. Circulation: Cardiovascular Interventions. 2009; 2(3): 159-66.

44. Halm EA, Tuhrim S, Wang JJ, Rockman C, Riles TS, Chassin MR. Risk factors for perioperative death and stroke after carotid endarterectomy: Results of the new york carotid artery surgery study. Stroke. 2009; 40(1): 221-9.

45. Hamdan AD, Pomposelli FB, Jr., Gibbons GW, Campbell DR, LoGerfo FW. Renal insufficiency and altered postoperative risk in carotid endarterectomy. Journal of Vascular Surgery. 1999; 29(6): 1006-11.

46. Hopkins LN, White CJ, Foster MT, Powell RJ, Zemel G, Diaz-Cartelle J. Carotid artery stenting and patient outcomes: The CABANA surveillance study. Catheter Cardiovasc Interv. 2014; 84(6): 997-1004.

47. Jalbert JJ, Nguyen LL, Gerhard-Herman MD, Jaff MR, White CJ, Rothman AT, et al. Outcomes after carotid artery stenting in Medicare beneficiaries, 2005 to 2009. JAMA Neurol. 2015; 72(3): 276-86.

48. Jim J, Dillavou ED, Upchurch GR, Jr., Osborne NH, Kenwood CT, Siami FS, et al. Gender-specific 30-day outcomes after carotid endarterectomy and carotid artery stenting in the Society for Vascular Surgery Vascular Registry. Journal of Vascular Surgery. 2014; 59(3): 742-8.

49. Kragsterman B, Bjorck M, Lindback J, Bergqvist D, Parsson H, Swedish Vascular R. Long-term survival after carotid endarterectomy for asymptomatic stenosis. Stroke. 2006; 37(12): 2886-91.

50. Kresowik TF, Bratzler DW, Kresowik RA, Hendel ME, Grund SL, Brown KR, et al. Multistate improvement in process and outcomes of carotid endarterectomy. Journal of vascular surgery. 2004; 39(2): 372-80.

51. Kucey DS, Bowyer B, Iron K, Austin P, Anderson G, Tu JV. Determinants of outcome after carotid endarterectomy. Journal of Vascular Surgery. 1998; 28(6): 1051-8.

52. Lindstrom D, Jonsson M, Formgren J, Delle M, Rosfors S, Gillgren P. Outcome after 7 years of carotid artery stenting and endarterectomy in Sweden - Single centre and national results. European Journal of Vascular and Endovascular Surgery. 2012; 43(5): 499-503.

53. Long GW, Nuthakki V, Bove PG, Brown OW, Shanley CJ, Bendick PJ, et al. Contemporary Outcomes for Carotid Endarterectomy at a Large Community-Based Academic Health Center. Ann Vasc Surg. 2007; 21(3): 321-7.

54. Lubke T, Ahmad W, Brunkwall J. Gender-based 30-day and long-term outcomes after carotid endarterectomy. Vasa - European Journal of Vascular Medicine. 2015; 44(4): 289-95.

55. Lutz HJ, Michael R, Gahl B, Savolainen H. Local versus general anaesthesia for carotid endarterectomy--improving the gold standard ? European Journal of Vascular & Endovascular Surgery. 2008; 36(2): 145-9; disussion 50-1.

56. Massop D, Dave R, Metzger C, Bachinsky W, Solis M, Shah R, et al. Stenting and angioplasty with protection in patients at high-risk for endarterectomy: SAPPHIRE Worldwide Registry first 2,001 patients. Catheter Cardiovasc Interv. 2009; 73(2): 129-36.

57. Mattos MA, Sumner DS, Bohannon WT, Parra J, McLafferty RB, Karch LA, et al. Carotid endarterectomy in women: challenging the results from ACAS and NASCET. Annals of Surgery. 2001; 234(4): 438-45; discussion 45-6.

58. Palombo D, Lucertini G, Mambrini S, Spinella G, Pane B. Carotid endarterectomy: Results of the Italian Vascular Registry. Journal of Cardiovascular Surgery. 2009; 50(2): 183-7.

59. Parlani G, De Rango P, Cieri E, Verzini F, Giordano G, Simonte G, et al. Diabetes is not a predictor of outcome for carotid revascularization with stenting as it may be for carotid endarterectomy. Journal of Vascular Surgery. 2012; 55(1): 79-88.

60. Pulli R, Dorigo W, Barbanti E, Azas L, Pratesi G, Innocenti AA, et al. Does the high-risk patient for carotid endarterectomy really exist? Am J Surg. 2005; 189(6): 714-9.

61. Reed AB, Gaccione P, Belkin M, Donaldson MC, Mannick JA, Whittemore AD, et al. Preoperative risk factors for carotid endarterectomy: Defining the patient at high risk. Journal of Vascular Surgery. 2003; 37(6): 1191-9.

62. Rockman CB, Halm EA, Wang JJ, Chassin MR, Tuhrim S, Formisano P, et al. Primary closure of the carotid artery is associated with poorer outcomes during carotid endarterectomy. Journal of Vascular Surgery. 2005; 42(5): 870-7.

63. Rockman CB, Maldonado TS, Jacobowitz GR, Cayne NS, Gagne PJ, Riles TS. Early carotid endarterectomy in symptomatic patients is associated with poorer perioperative outcomes. J Vasc Surg. 2006; 44(3): 480-7.

64. Schreiber TL, Strickman N, Davis T, Kumar V, Mishkel G, Foster M, et al. Carotid artery stenting with emboli protection surveillance study: outcomes at 1 year. Journal of the American College of Cardiology. 2010; 56(1): 49-57.

65. Sedivy P, El Samman K, Prindisova H, Stadler P. The importance of and current trends in the endovascular program - A single center experience. Cor et Vasa. 2015; 57(2): e101-e7.

66. Setacci C, Chisci E, Setacci F, Iacoponi F, De Donato G, Rossi A. Siena carotid artery stenting score: A risk modelling study for individual patients. Stroke. 2010; 41(6): 1259-65.

67. Sidawy AN, Aidinian G, Johnson ON, 3rd, White PW, DeZee KJ, Henderson WG. Effect of chronic renal insufficiency on outcomes of carotid endarterectomy. Journal of Vascular Surgery. 2008; 48(6): 1423-30.

68. Stabile E, Salemme L, Sorropago G, Tesorio T, Nammas W, Miranda M, et al. Proximal Endovascular Occlusion for Carotid Artery Stenting. Results From a Prospective Registry of 1,300 Patients. Journal of the American College of Cardiology. 2010; 55(16): 1661-7.

69. Stabile E, Garg P, Cremonesi A, Bosiers M, Reimers B, Setacci C, et al. European registry of carotid artery stenting: Results from a prospective registry of eight high volume EUROPEAN institutions. Catheterization and Cardiovascular Interventions. 2012; 80(2): 329-34.

70. Stromberg S, Gelin J, Osterberg T, Bergstrom GM, Karlstrom L, Osterberg K, et al. Very urgent carotid endarterectomy confers increased procedural risk. Stroke. 2012; 43(5): 1331-5.

71. Tu JV, Wang H, Bowyer B, Green L, Fang J, Kucey D. Risk Factors for Death or Stroke After Carotid Endarterectomy: Observations From the Ontario Carotid Endarterectomy Registry. Stroke. 2003; 34(11): 2568-73.

72. Waton S, Johal A, Groene O, Cromwell D, Mitchell D, Loftus I. UK carotid endarterectomy audit. Round 5. 2013.

73. Munster AB, Franchini AJ, Qureshi MI, Thapar A, Davies AH. Temporal trends in safety of carotid endarterectomy in asymptomatic patients Systematic review. Neurology. 2015; 85(4): 365-72.

74. Vikatmaa P, Mitchell D, Jensen LP, Beiles B, Bjorck M, Halbakken E, et al. Variation in clinical practice in carotid surgery in nine countries 2005-2010. Lessons from VASCUNET and recommendations for the future of national clinical audit. European Journal of Vascular and Endovascular Surgery. 2012; 44(1): 11-7.

75. Wholey MH, Al-Mubarek N, Wholey MH. Updated review of the global carotid artery stent registry. Catheter Cardiovasc Interv. 2003; 60(2): 259-66.

76. Wu TY, Akopian G, Katz SG. Patients at elevated risk of major adverse events following endarterectomy for asymptomatic carotid stenosis. Am J Surg. 2015; 209(6): 1069-73.

77. Yang SS, Kim YW, Kim DI, Kim KH, Jeon P, Kim GM, et al. Impact of contralateral carotid or vertebral artery occlusion in patients undergoing carotid endarterectomy or carotid artery stenting. Journal of Vascular Surgery. 2014; 59(3): 749-55.

78. Yoshida S, Bensley RP, Glaser JD, Nabzdyk CS, Hamdan AD, Wyers MC, et al. The current national criteria for carotid artery stenting overestimate its efficacy in patients who are symptomatic and at high risk. Journal of Vascular Surgery. 2013; 58(1): 120-7.
